# Ionic Liquid‐Based Polymer Nanocomposites for Sensors, Energy, Biomedicine, and Environmental Applications: Roadmap to the Future

**DOI:** 10.1002/advs.202202187

**Published:** 2022-07-19

**Authors:** Kirti Mishra, Nishu Devi, Samarjeet Singh Siwal, Qibo Zhang, Walaa F. Alsanie, Fabrizio Scarpa, Vijay Kumar Thakur

**Affiliations:** ^1^ Department of Chemistry M.M. Engineering College Maharishi Markandeshwar (Deemed to be University) Mullana‐Ambala Haryana 133207 India; ^2^ Mechanics and Energy Laboratory Department of Civil and Environmental Engineering Northwestern University 2145 Sheridan Road Evanston IL 60208 USA; ^3^ Key Laboratory of Ionic Liquids Metallurgy Faculty of Metallurgical and Energy Engineering Kunming University of Science and Technology Kunming 650093 P. R. China; ^4^ State Key Laboratory of Complex Nonferrous Metal Resources Cleaning Utilization in Yunnan Province Kunming 650093 P. R. China; ^5^ Department of Clinical Laboratories Sciences The Faculty of Applied Medical Sciences Taif University P.O. Box 11099 Taif 21944 Saudi Arabia; ^6^ Bristol Composites Institute University of Bristol Bristol BS8 1TR UK; ^7^ Biorefining and Advanced Materials Research Center Scotland's Rural College (SRUC) Kings Buildings, West Mains Road Edinburgh EH9 3JG UK; ^8^ School of Engineering University of Petroleum and Energy Studies (UPES) Dehradun Uttarakhand 248007 India

**Keywords:** actuators, biomedicine, energy storage materials, fuel cells, ionic liquids‐based polymer nanocomposite, ionogels, sensors

## Abstract

Current interest toward ionic liquids (ILs) stems from some of their novel characteristics, like low vapor pressure, thermal stability, and nonflammability, integrated through high ionic conductivity and broad range of electrochemical strength. Nowadays, ionic liquids represent a new category of chemical‐based compounds for developing superior and multifunctional substances with potential in several fields. ILs can be used in solvents such as salt electrolyte and additional materials. By adding functional physiochemical characteristics, a variety of IL‐based electrolytes can also be used for energy storage purposes. It is hoped that the present review will supply guidance for future research focused on IL‐based polymer nanocomposites electrolytes for sensors, high performance, biomedicine, and environmental applications. Additionally, a comprehensive overview about the polymer‐based composites’ ILs components, including a classification of the types of polymer matrix available is provided in this review. More focus is placed upon ILs‐based polymeric nanocomposites used in multiple applications such as electrochemical biosensors, energy‐related materials, biomedicine, actuators, environmental, and the aviation and aerospace industries. At last, existing challenges and prospects in this field are discussed and concluding remarks are provided.

## Graphical abstract

In this study, ionic liquids based polymer composites and their exceptional physicochemical characteristics and their capability to bond by analytes via multiple interchanges, such as *π*–*π*, hydrogen interaction, ion‐exchange, electrostatic, and dipolar relations, are discussed in detail.

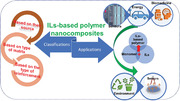

## 1 Introduction

The discovery of water and air‐stable ionic liquids (ILs) by Wilkes in 1992 has opened a new field of interest within the advanced multifunctional materials community. During the last 20 years, more focus has been paid to ILs because of their novel and adjustable physical and chemical features.^[^ ^1^ ^]^ ILs are mainly represented as an electrolyte that is liquid and made up of ions, and has a melting point of less than 100 °C. These characteristics differentiate ILs from molten salts.^[^ ^2^ ^]^ ILs are constituted by various organic cations, and anions that are organic or inorganic. ILs show unique properties depending on their various configurations, based on hydrogen bonding, columbic forces, and van der Waals interactions between ions, i.e., cations and anions.^[^ ^3^ ^]^ Ionic liquids also possess a moderate vapor pressure, which is lower than their decomposition temperature. The majority of the ILs compounds are liquid at 20 °C, i.e., at room temperature.^[^ ^4^ ^]^

The classical configurations of ILs in ambient condition or beneath ≤100 °C can assume fused, molten, and liquids organic salts.^[^ ^5^ ^]^ Few ILs‐based polymers are supposed to be exclusively ionic, while being defined as deep eutectic solvents (DESs) and protic ILs (PILs) that are rare with the reagent of Olah's.^[^ ^6^ ^]^ Furthermore, ILs have a significant lower viscosity and vapor pressure, and they may not vaporize easily beneath room temperature. ILs are also soluble in various solvents, can be acid and base, have wide‐range temperature steadiness, and are much less prone to corrosion than inorganic acids and base*s*.^[^ ^7^ ^]^ Because of their nominal vapor pressure, ILs do not describe the explorer threat compared to ignitable organic solvents. ILs also do not show any harmful effect on the photochemistry of the environment. Furthermore, the essential lack of vaporization in ILs provides nonflammability below room temperatures; this includes some solvents with a lower boiling point, for example, pet ether, dichloromethane acetone, etc.^[^ ^8^ ^]^

Structure and functions of ionic liquids have been previously discussed in scholarly works related to the characterization of ILs.^[^ ^9^ ^]^ Focus has also been placed about the descriptions of distinct functionalities,^[^ ^10^ ^]^ ILs with chiral center,^[^ ^11^ ^]^ ILs conversion of a solvent's polarity,^[^ ^12^ ^]^ bio‐based‐ILs,^[^ ^13^ ^]^ polymerized ILs,^[^ ^14^ ^]^ active ILs,^[^ ^15^ ^]^ unbiased ILs,^[^ ^16^ ^]^ protic ILs,^[^ ^17^ ^]^ metal ILs,^[^ ^18^ ^]^ base ILs,^[^ ^19^ ^]^ sustainable ILs,^[^ ^20^ ^]^ and ILs preparation routes. Herein the presented review, we try to collate a comprehensive list of details related to the general overview of polymer‐based composites’ ILs components, including a classification of the polymer matrix. Furthermore, more focus is placed on ILs‐based polymeric nanocomposites used in multiple applications (such as electrochemical biosensors, energy related materials, biomedicine, actuators, environmental and aviation and aerospace industries). We then finally discuss existing challenges and prospects in the ILs field.

## 2 General Overview of Ionic Liquids

ILs are usually described as compounds comprised of cations and anions having melting temperature under 100 °C. Paul Walden reported the initial IL (ethyl ammonium nitrate) in 1914, and ILs were not recognized as a significant scientific field until after nearly one century.^[^ ^21^ ^]^ By providing atmospheric condition, in 1940, at the Rice Institute of Texas, two scientists, Frank Hurley and Tom Weir designed liquid salts. When a salt of alkyl pyridinium chloride was mixed with aluminum chloride (AlCl_3_) and converted into a transparent liquid, it was called an ionic liquid. Their finding about ILs remained a chemical curiosity until the following years.^[^ ^22^ ^]^

**Figure 1:**
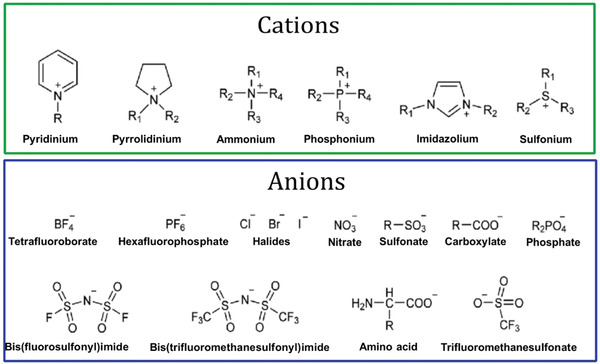
Some common cations and anions that form ILs. Reproduced with permission.^[^ ^23^ ^]^ Copyright 2021, MDPI.

**Figure 2:**
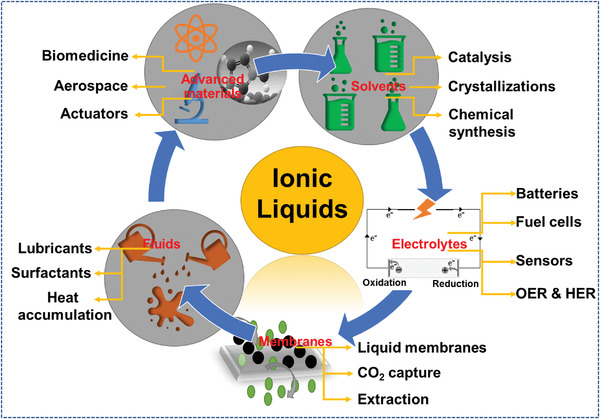
The role of ILs in different fields of research.

ILs are liquids prepared from cations and anions. Some general examples of cations and anions have been shown in **Figure** **1**. These cations and anions are generally connected via different types of bonds. They are related to multiple physical and chemical characteristics, with deficient to nearly negligible vapor pressure and adequate thermal strength. In previous years, major studies dedicated to ILs highlight the growth of green and sustainable chemistry. Related fundamental and applied investigations have grown exponentially and have also shown the ILs importance to science, including a broad range of possible applications (**Figure** **2**). ILs are especially known as green solvents and/or composites that can replace traditional flammable organic solvents.

**Figure 3:**
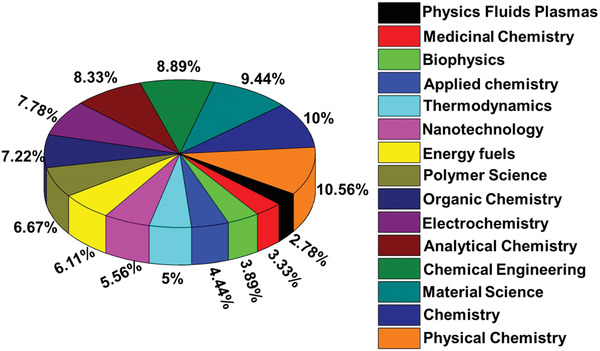
Classification of fields of interest and applications versus the number of publications. Adapted with permission.^[^ ^36a^ ^]^ Copyright 2020, Elsevier Ltd.

ILs as paradigmatic new classes of fluids have attracted significant attention only during the previous two decades. The number of science citation index (SCI) articles published on ILs has exponentially grown from isolated articles, to more than 5000 between 1996 and 2016; the yearly rates of growth of papers about ILs has surpassed those of other popular scientific topics. This suggests that more and more investigators are involved in the exploration of this exciting field. The investigation of ILs is a multidisciplinary endeavor that involves analytical chemistry,^[^ ^24^ ^]^ materials science,^[^ ^25^ ^]^ chemical engineering,^[^ ^26^ ^]^ and environmental science.^[^ ^27^ ^]^ Furthermore, ILs show clear advantages for the preparation, catalysis,^[^ ^28^ ^]^ physical chemistry,^[^ ^29^ ^]^ electrochemistry,^[^ ^30^ ^]^ energy fuels,^[^ ^31^ ^]^ heredity,^[^ ^32^ ^]^ nuclear physics,^[^ ^33^ ^]^ medicinal chemistry,^[^ ^34^ ^]^ engineering,^[^ ^35^ ^]^ at manufacturing and laboratory scales. **Figure** **3** shows that the primary emphasis of the fields of interest of ILs is centered on material science, physical chemistry, chemical engineering, and multipurpose chemistry. At the same time, they are dominant over different implementations of ILs. Due to their ongoing development, it might be helpful to manage ILs physicochemical effects and many more characteristics of significance.^[^ ^36^ ^]^

ILs are liquids prepared from cations and anions. These ions are generally connected via different kinds of bonds. They are related to multiple physical and chemical characteristics, with deficient to nearly inconsequential vapor pressure, adequate thermal strength, and many more may be utilized as a green solvent and/or composite.

### 2.1 IL‐Based Polymer Composite

ILs constitute a new category of chemical substances for superior (multi)purpose materials, with excellent prospectives of implementation in numerous fields because of their distinctive properties and functions. The introduction of various other substances can customize the properties of the ILs; one of them is polymers. The amalgamation of ILs with a particular type of polymers opens the opportunity to increase new substances having distinct structure such as membrane, films, fibers, etc. for the stimuli‐responses with customization, as a novel proposal for highly developed multifunctional materials. Moreover, the relationship between the chosen IL charges, i.e., cations and anions, also provides a significant functionality to the composite materials when combined with the polymer chains. The amalgamation of various ILs with different polymers opens new areas of applications in the field of sensors, drug delivery, actuators, batteries and fuel cells, environment, and biomedical. The functionalities of ILs can also be instrumental in enhancing the properties of future generations of composite structural batteries; ILs composites/nanocomposites could be integrated within the battery's architecture to increase energy density and tuning of the dielectric properties. For example, an IL, namely, diethyl methylammonium trifluoromethanesulfonate ([dema][TfO]) mixed with a polymer, i.e., polybenzimidazole and an IL‐based polymer electrolytes for fuel cells fabricated having operating temperatures above 100 °C. The membrane shows better thermal stability, high proton conductivity, i.e., 108.9 mS cm^−1^ at 250 °C and higher conductivity stability.^[^ ^37^ ^]^ Further, an IL‐based ionic electroactive polymer (IL‐iEAP) transducer design for highly developed applications in biological and electronic areas. The advanced functionality of this IL‐iEAP is in 3D‐printing.^[^ ^38^ ^]^ A study shows that when alkyl imidazolium‐ and alkyl phosphonium‐based IL incorporated with biodegradable polymers composite (BPC), i.e., polylactic acid, polybutylene succinate, and polycaprolactone, which all enhance the thermal, chemical, and mechanical properties of polymers composites. Alkyl imidazolium‐based ILs provide BPC a prominent elongation quality, excellent thermal stability, and superior compatibility. On the other hand, alkyl phosphonium‐based ILs provide BPC with a better tensile loading capacity, superior thermal stability, and better multiphysics interaction.^[^ ^3c^ ^]^ Fan et al.^[^ ^39^ ^]^ reported Ti_3_C_2_–MXene blended layer functionalized with polypyrrole and IL‐based microemulsion fragments for supercapacitor applications. The above‐fabricated film shows outstanding specific capacitance, rate capability as well as cycling stability in between 4 and 50 °C. Further, the highest gravimetric density of 31.2 Wh kg^−1^ (at 1030.4 Wh kg^−1^) was exhibited by the fabricated film at room temperature for devices that are based on symmetric super capacitors.

### 2.2 Fabrication and Properties of IL‐Based Polymer Composites

This section summarizes the recent strategies for the fabrication process of IL‐based polymer composites, such as in situ polymerization, melt mixing, solution blending, and the use of ILs as filler to the polymer matrix.

A facile and environmentally friendly route of IL‐based polymer nanocomposites fabrication involved the use of 1‐butyl‐3‐methylimidazolium bromide as ionic liquid and polyaniline as polymer matrix and was synthesized using in situ polymerization and a cationic surfactant CTAB. This method helps retain the composite's conductivity with a frequency 3 MHz and temperature 120 °C.^[^ ^40^ ^]^ Another example of one‐pot in situ polymerization is synthesis of reduced graphene oxide (rGO)@poly(ionic liquide) (PIL)/PBO where 1‐vinyl‐3‐aminopropylimidazole tetrafluoroborate (IL) converted to poly(1‐vinyl‐3‐aminopropyl imidazolium ionic liquid) (PIL‐NH_2_) by in situ free radical polymerization where PIL attached to rGO by *π*–*π* conjugation with improved dielectric properties, thermal stability, and high energy density.^[^ ^41^ ^]^ Shamsuri et al.^[^ ^3c^ ^]^ provided a review on the employment of ILs as fillers that form a biodegradable polymer composite. Such ILs‐based biodegradable polymer composites were found to exhibit dual benefits, including improvement in properties and environment‐friendly polymer matrix.

As we know, ILs are composed of different cations and anions. The selection of these during the preparation of ILs‐based polymeric nanocomposites plays a crucial role. Apart from the type of polymer matrix, ILs compositions like the size of ions, length of an alkyl chain, type of anion, and interaction between the ions could also affect the properties and hence application of the ILs‐based polymer composites. For example, ILs based on ammonium, imidazolium, and phosphonium cations could be an alternative to traditional plasticizers of PVC because of their diffusion rate. The size and molecular structure of cations and anions in an IL contribute to the diffusion rate, intermolecular interactions, and hence localization to the polymer matrix. The thermal stabilities of some of the anions (bis(trifluoromethanesulphonyl) imide, TFSI)− > (PF_6_) − >> (Br)− also provides a pathway to select the anion for a thermal application. In addition, the length of the alkyl chain could also participate in affecting the properties. Fedosse Zornio et al.^[^ ^42^ ^]^ studied and compared the chemical nature of 1‐butyl‐3‐methylimidazolium, *N*‐trimethyl‐*N*‐butyl ammoniun, *N*‐trimethyl‐*N*‐hexyl ammonium as cations and bromide, hexafluorophosphate, bis(trifluoromethanesulfonyl)imide as anions in poly‐1‐(methyl methacrylate) (PMMA) polymer matrix.

**Figure 4:**
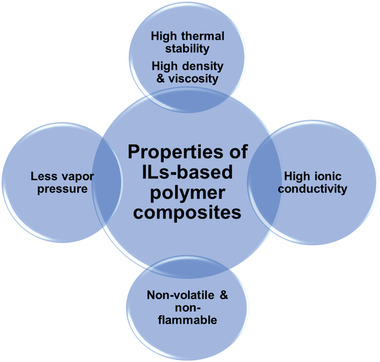
Properties of ionic liquid‐based polymer.

One of the fundamental limitations of IL polymers is the reduced ionic conductivity at ambient temperatures. One of the suggestions provided in literature to deal with this is developing IL polymer at lower glass transition temperature that could help retain ion mobility and, hence, conductivity. One such analysis on the size of ions and mobility is carried out by Stacy et al.,^[^ ^43^ ^]^ where they discussed how columbic and elastic forces contribute to the ion transport in poly‐ILs and how the size of ions affects the diffusion. **Figure** **4** shows the different properties of the ionic liquid‐based polymer.

## 3 Component and Classification of Polymer Composites

### 3.1 Component of Polymer Composites

A requisite for economical and lightweight materials has resulted in extreme curiosity in polymers in the past few years.^[^ ^44^ ^]^ Polymer‐based composites are polyphase compounds where different types of fillers can be reinforced inside the polymer matrix, emerging in harmonious mechanical nature which is unattainable by using polymer alone.^[^ ^45^ ^]^ Polymer‐based composites are usually based on two components, namely, fiber and matrix.^[^ ^46^ ^]^

#### 3.1.1 Fiber

Fibers, natural or synthetic, are high strength/high modulus materials. Examples of natural fibers are reinforcements extracted from jute, oil palm, sisal, date palm, hemp, and flax.^[^ ^47^ ^]^ Typical fossil fibers are represented by glass materials, Kevlar, carbon fiber, and polyethylene.^[^ ^48^ ^]^ The orientation and arrangement of a fiber define the composite material's structural behavior and properties.^[^ ^49^ ^]^ Hybrid composites can be obtained by blending these two classes of fibers with any matrix material.^[^ ^50^ ^]^

**Figure 5:**
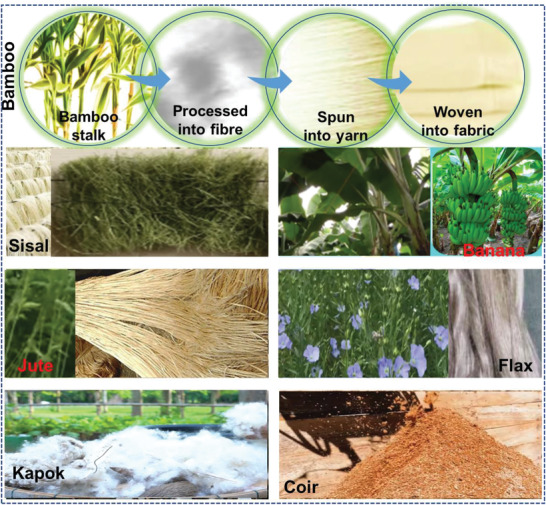
Some common examples of natural fibers.

Numerous investigators have documented the benefits of cellulosic fibers: the latter are functional, nontoxic, renewable, low‐cost, and provide essential bonding with the matrix for load‐transfer capabilities that enhance flexibility and toughness under bending, all properties that affect the structural resistance.^[^ ^51^ ^]^ Currently, limestone powder, fly ash, brick powder, and numerous types of mineral stabilizers support the microstructure architectures of the composites.^[^ ^52^ ^]^ Enhancement of failure resistance and stiffness have been improved by means of the use of fly ash within a composite for load‐bearing capabilities, which results in an increased lifetime of the composite materials. Asbestos present within fibers is unsafe for human's health; asbestos‐based reinforcements are therefore not much explored in fiber‐reinforced composite materials. Plant‐based fibers deliver practical aspects like cost‐effectiveness, biodegradability, accessibility, and better physical and self‐regulating characteristics.^[^ ^53^ ^]^ Plant‐based fibers have leaf (sisal and abaca), bast (flax, jute, hemp, ramie, and kenaf), fodder and reed (rice husk), core (hemp, jute, and kenaf), seed (cotton, kapok, and coir), and all other types of fibers originated from timber and wood.^[^ ^54^ ^]^ **Figure** **5** displays some common examples of natural fibers.

#### 3.1.2 Matrix

The matrix can be a thermoset polymer like polydicyclopentadiene, epoxy resin, or polyimide or can be a thermoplastic like polystyrene (PS), polypropylene (PP), acrylic, polyesters, and epoxy resins be elastomer type.^[^ ^55^ ^]^ The function of the matrix is to form a connection with fibers and avail a reinforcement and desired structure to the final composite. The characteristics of the matrix decide the resistance power of the polymer composites to impact damage, attack of chemicals, high‐temperature creep, and water absorption.^[^ ^56^ ^]^ There are various thermoplastic matrices with a broad scope of refreshing characteristics. Due to its lightweight, excessive mechanical power and protection from the adverse consequence of the environment, in various applications, thermoplastics are used as absolute substances.

### 3.2 Classification of ILs‐Based Polymer Composites

**Figure 6:**
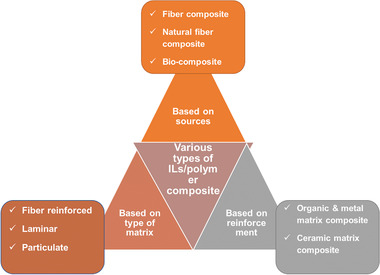
Classification of ILs‐based polymer composites.

Based on the source, the type of reinforcement and type of matrix polymer composites are classified. **Figure** **6** shows the classification of ILs‐based polymer composites.

#### 3.2.1 Classification Based on Their Source

##### Fiber Composites

Fiber‐reinforced composites are high performing fiber‐based composites and are composed of cross‐linked cellulose‐based fiber molecules, which include resins in the fiber‐reinforced composite matrix by a patented molecular re‐engineering phenomenon, resulting in an invention of excellent structural characteristics. With the aid of molecular re‐engineering, wood's desirable material, and structural characteristics are positively cloned and clad in the fiber‐reinforced composite product, that yield more significant characteristics and performance than the existing wood.^[^ ^57^ ^]^ Fiber‐reinforced composite materials, unlike other composites, can be recycled 20 times, allowing scrap and can be reused multiple times.

##### Natural Fiber Composites

These are the materials in which the fibers reinforced in nature are made up of some substances like plants and wood that act as renewable resources for carbon dioxide. Their environmental and biological durability is frequently quoted as a critical benefit to them compared to conventional materials. Natural fibers have received special attention from engineers, technologists, manufacturers, and industrialists due to their vast prospective and applications in various engineering functions in the railway, building construction, automotive, defense, packaging, and general secondary‐load bearing applications.^[^ ^58^ ^]^ Also, natural fiber composites are cost‐effective, renewable materials that contribute to reduce CO_2_ and other emissions, and are environmentally friendly.^[^ ^59^ ^]^

##### Biocomposites

These are composite materials made up of matrix (resin) and reinforcement of natural fibers.^[^ ^60^ ^]^ Biocomposites frequently mimic the living materials structure involved in the process used for maintaining matrix strengthening properties by always providing biocompatibility. The matrix part is derived with polymers like—for example—polyester and natural rubber. Those polymers are obtained from renewable and nonrenewable resources.^[^ ^61^ ^]^ Biofibers are divided into two parts, i.e., wood and nonwood fibers, all of which present lignin and cellulose. Fibers are crucial elements in the design of biocomposites and can be obtained from biological sources like animals or plants (e.g., fibers from recycled wood, crops like cotton, flax, or hemp, desecrate paper, regenerated cellulose fiber (viscose/rayon), silk or crop processing byproducts). Biocomposites are promising materials for potential future applications in the automotive and—more generally—the transport, apparel and packaging industries. Their attractive features, i.e., sustainability and renewability and further being biodegradable, make biocomposites a “material of the future.” Easy to dispose of and because of organic fertilizers characteristic of biocomposites later than the expiration is significant advantages of biocomposites, this is not usually possible with traditional synthetic substances.^[^ ^62^ ^]^

#### 3.2.2 Classification Based on the Type of Matrix

Depending upon the type of matrix, polymer composites are classified into three types.

##### Organic Matrix Composites

It comprises numerous short and continuous fibers bound collectively via an organic polymer matrix. These composites are usually made for load among fibers of a matrix. They are lightweight, highly stiff, and has high strength and reinforcement.^[^ ^63^ ^]^ Basis of used polymer composite material are classified into three categories.

###### Thermoplastic Composites

Thermoplastic resins commonly used in composite materials are typically high‐density polyethene, PP, poly(vinylidene fluoride) (PVDF), and polylactic acid.^[^ ^64^ ^]^ Due to their higher viscosity, they are less used as a high‐tech material because of problems related to the effective wetting of the reinforcement during thermoforming, with the formation of porosity. The thermoplastic matrices are arranged in straight chains that can change the melted form. Generally, they are prepared by heating after shaping, injection, taking out, or thermoforming earlier than cooling. In this way, the final output retains the initially designed configuration,^[^ ^65^ ^]^ and this process can be reverted. Thermoplastic matrices are resistant to oxidation and corrosion with good electrical and thermal insulating properties; they are used in many applications as ideal materials because of their excellent properties like lightweight and resistance to environmental effect.

The thermoplastic matrices are present in the shape of unbent chains that may be altered within the melted form. In highly automated manufacturing processes, the thermoplastic matrices are warmed then assembled with injection moulding, extraction or early thermoforming. This process is reversible. Reversibility is important, because it makes thermoplastic matrices recyclable, with a significant positive impact to the life cycle of the products and the overall global warming potential of composites made with those matrices. There are numerous thermoplastics, including a broad range with exciting characteristics.^[^ ^66^ ^]^ Those matrices can be fashioned elastic like rubber, tough like metal and concrete, or translucent like glass to obtain multiple functionalities. Those matrices do not oxidize, have an elevated protection from deterioration, and offer superior insulation for heat and electricity. Owing to their lightweight, mechanical stability, and resistance to environmental challenges, thermoplastics are excellent materials for a wide variety of uses.

###### Thermoset Composite

Thermoset polymer composites consist of unsaturated polyester epoxy vinyl ester that acts as resins. They are primarily used in automotive, aerospace, naval, and aeronautical applications.^[^ ^67^ ^]^ They are liquid at room temperature and can be solidified during implementation of warm, and an additional material known as hardener. In this way they are converted by heating, a type of chemical change‐making 3D strong bond. This is a permanent change and cannot be reversed. Due to this process, the material becomes insoluble in solvents like alcohols, hydrocarbons, ketones, etc. They are harder than thermoplastic matrices, crease‐resistance, and better‐woven fibers.

Typically liquid on ambient conditions, thermosets harden when subjected to heat and a hardener stabilizer. Therefore, curing affects the behavior of thermosets, inducing a chemical conversion with three different types of bonds among molecules. This operation is permanent, and once the material is melted is not soluble anymore within the mix of solvents (such as ketones, alcohols, and hydrocarbons). These materials are stiffer compared to thermoplastic matrices and are appropriate for moulding/resin infuse reinforcements with small, long or woven fibers.^[^ ^68^ ^]^ The most common used thermoset matrices are polyesters, phenoplasts, epoxy resins, polyurethanes (PUs), phenolic, polymides, bis‐maleimides, and silicones.

###### Elastomers

Elastomer polymers have the same flexible grades of natural rubber. An inactive elastomer includes extended chains of molecules that are joined with each other one by one. The molecules of elastomers may have relative motions relative to each other and distort. For better elastic properties, the base materials are vulcanized. Vulcanization makes the elastomer hard, and helps forming a better or slightly more rigid 3D structure without major detriment to the elasticity of the molecular chains. Sulfur, carbon, and diverse chemically active compounds are present within an elastomer.^[^ ^69^ ^]^ Various formulations permit the development of artificial rubbers for different applications. Elastomers are used to manufacture pillows, specific insulators, shoe soles or tires.

##### Metal Matrix Composites

Metal‐based fibers are reinforcements with specific high‐toughness. They are generally used for the strengthening of metal matrices via stacking and alternate bonding. Their demand is low because of their high cost.^[^ ^70^ ^]^ To resist high temperatures, i.e., 300 °C of metal matrices, carbon‐steel fibers are used. Some other commonly used heat resistance metal fibers are tungsten, molybdenum, and boron. Metal matrix composites require reinforcements that are stable over a wide range of temperatures, and chemically inert. Due to an increase of the melting point of the matrix material, their reinforcements becomes small.^[^ ^71^ ^]^

##### Ceramics Matrix Composites (CMCs)

CMCs are technical ceramics, as well as composite materials. They are designed with ceramic fibers embedded in a ceramic‐based matrix.^[^ ^72^ ^]^ The single phases of CMCs are brittle, To overcome the disadvantage of monolithic ceramics (i.e., their fragility), CMCs are developed. CMCs are rigid materials and show potential failure behavior during FM bonding. Improvement of the bonding between the reinforcement and the ceramic matrix is generally obtained by using a fiber coating, known as interphase.^[^ ^73^ ^]^ CMCs are used primarily in applications where resistance to high temperatures and corrosive environments are required. They lack toughness but are quite strong and stiff, although CMC with architected reinforcements can provide significant resistance to high‐temperature creep, even at low volume fractions of reinforcement. Matrix materials mainly used in CMCs are silicon carbide, nitrides, mullite, and aluminum oxide, whose strength is maintained up to 1649 °C. They are generally used in automobile engines, cutting tools, mining, and spacecraft applications.

##### Copolymer‐Based Matrix

**Figure 7:**
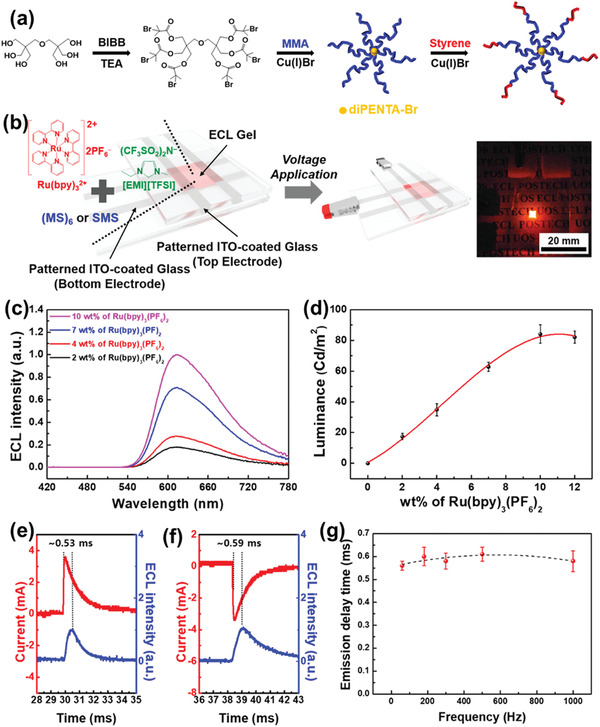
a) Diagrammatic representation of the preparation of star‐shaped block copolymers (MS)_6_. b) Schematic description and picture of the ECL apparatus, including an emissive gel coating comprised of Ru(bpy)_3_(PF_6_)_2_/(MS)_6_/[EMI][TFSI] within the weight proportion of 1:3:7. The size of the individually emitting region was 2 × 2 mm. c) Emission graph from ECL appliances at different Ru(bpy)_3_(PF_6_)_2_ absorptions from 2 to 12 wt% comparative to the part of [EMI][TFSI]. The band at *λ*_max_ = 610 nm (2.04 eV) approximates to emission of Ru(bpy)_3_^2+^ from the ^3^MLCT higher energy band. d) Graphs of luminance as a part of Ru(bpy)_3_(PF_6_)_2_ absorption beneath a used AC peak‐to‐peak potential (*V*_pp_) of 6.6 V (i.e., −3.3 to +3.3 V) on a frequency of 60 Hz. The reaction period swapped from e) anode to cathode and f) cathode to anode bias. g) Graphs of the reaction period at different frequencies. Reproduced with permission.^[^ ^74^ ^]^ Copyright 2019, American Chemical Society.

At present polymer‐based gel electrolyte seeks much attention due to its much electrochemical applications. Further, the structure of polymer gelators is helpful for understanding the physical characteristics of ionogels (IGs). So that recently designs of these polymers attracted much interest for the enhancement in the mechanical and conductive properties of materials. Gelators are present in two forms one is homopolymers, and another is copolymers. A triblock copolymer is a good host material for mechanical robustness out of these two versions of polymers. In a triblock copolymer, both end blocks are insoluble in short ILs. Upon mixing, they aggregated in the form of hard spherical domains and increased the IGs mechanical properties. On the other hand, ILs‐soluble midblock of copolymer on mixing with IL makes the ionic conductivity robust and hence initiates the electrochemical process. In this direction, a star‐shaped copolymer was fabricated by some researchers (poly(methyl methacrylate)‐*b*‐polystyrene)_6_ ((MS)_6_), which acts as a polymer gelator host for polymer‐based gel electrolyte. The six‐armed copolymer exhibits enhanced mechanical characteristics, approximate elasticity ≈2.5 × 10^4^ Pa, and better ionic conductivity ≈1.54 mS cm^−1^ at room temperature. Thermal stability also increases by using this star‐shaped copolymer (**Figure** **7**a).^[^ ^74^ ^]^

Figure 7b illustrates a graphic chart of the electrochemiluminescent (ECL) apparatus established upon (MS)_6_ gels. They set the emissive gel between a couple of line‐patterned indium tin oxide (ITO) electrodes, which obtained four emitting areas. Figure 7c illustrates the ECL emission ranges on Ru(bpy)_3_^2+^ concentrations. There was no support for the color of the emitted light upon the absorption of luminophores. It could be an emitted red‐orange light, including the highest wavelength of 610 nm at all concentrations transformed to 2.04 eV, conforming to Ru's triplet metal‐to‐ligand charge transfer (^3^MLCT) of Ru(bpy)_3_^2+^. Figure 7d illustrates the reliance of luminance upon the absorption of Ru(bpy)_3_^2+^. It marked the luminance saturation at 10 wt% of Ru(bpy)_3_^2+^, an inadequate arrangement with additional ion‐gel‐based ECL devices, including Ru(bpy)_3_^2+^. In principle, electrical double layers are assembled, and redox reactions appear about the electrodes when an exterior AC voltage is used. Then, excited luminophores are built through an electron transfer among redox species (i.e., Ru(bpy)_3_^•1+^ and Ru(bpy)_3_•^3+^). These operations carried site within ≈0.5 ms irrespective of preference polarization (Figure 7e,f), which corresponds to the reaction period of this appliance. While it varied the used frequency, there was no apparent deviation in the hold duration (Figure 7g).

**Figure 8:**
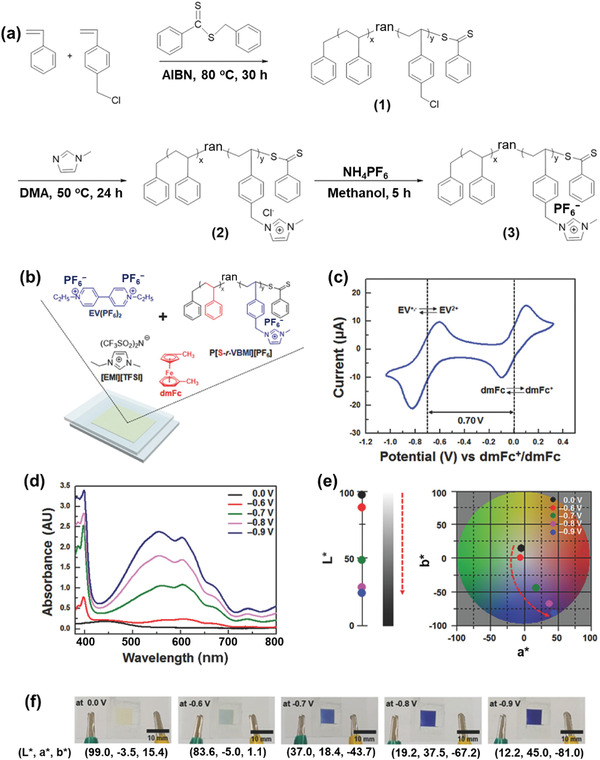
a) Synthetic pathway of poly[styrene*‐ran‐*1‐(4‐vinylbenzyl)‐3‐methylimidazolium hexafluorophosphate]. b) Molecular configurations of segments contained within the EC gel. c) CV of the EC gel, where the dmFc acts as an anodic ingredient and an internal measure. A Pt probe, ITO‐doped glass, and Ag‐wire were utilized as the working, counter, and quasi‐reference electrodes. Potential support of d) UV–vis absorption ranges and e) CIELAB color coordinates (L*a*b*) toward the electrochromic device (ECD), and f) photos of the ECD at different used potentials. Reproduced with permission.^[^ ^75^ ^]^ Copyright 2018, Wiley‐VCH.

Furthermore, by using a reversible addition–fragmentation chain transfer polymerization approach, a conductive IGs can be fabricated by using an IL 1‐ethyl‐3‐methylimidazolium bis(trifluoromethylsulfonyl)imide and a copolymer (random) poly[styrene*‐ran‐*1‐(4‐vinylbenzyl)‐3‐methylimidazoliumhexafluorophosphate]. The fabricated materials show better elasticity of 0.105 MPa and good ionic conductivity of 1.15 mS cm^−1^. **Figure** **8**a shows synthetic pathway of poly[styrene*‐ran‐*1‐(4‐vinylbenzyl)‐3‐methylimidazolium hexafluorophosphate].^[^ ^75^ ^]^

Figure 8b shows a schematic graphic of gel‐based electrochromic appliances and the chemical configurations of gel ingredients. Here, ethyl viologen bis(hexafluorophosphate) (EV(PF_6_)_2_) and dmFc were mixed within a gel comprised of P[S*‐r‐*VBMI][PF_6_] and [EMI][TFSI] as an electrochromic (EC) chromophore and anodic species. Figure 8c shows a cyclic voltammogram (CV) of the EC gel, where the redox responses of EV^2+^/EV^+•^ and dmFc^+/^dmFc ensued reversibly. Depending upon the start potential of dmFc oxidation (−0.07 V) and EV^2+^ reduction (−0.65 V), it is anticipated that the device coloration to start around ≈0.6 V. UV–vis absorption ranges were documented to study the potential reliance of the optical characteristics of the apparatus (Figure 8d). Without effective absorption, a potential window less than −0.6 V was marked, excluding an expansive and weak peak at ≈450 nm originating from the dmFc. As expected from the CV investigation, it produced colored EV^+•^ components around −0.6 V, and two firm absorption rises at ≈604 and ≈554 nm occurred. These distinct peaks became more assertive as the used potential improved due to the more elevated EV+ absorption of EV^+•^. It also tracked the optical variation concerning the CIELAB color coordinates (L*, a*, b*) (Figure 8e) and optical pictures (Figure 8f). Around 0.0 V, the L*, a*, and b* values ≈99.0, −3.5, and 15.4, indicating a little yellowish color because of the dmFc within EC gel. Nevertheless, a surprising decline within b* was noticed as the used potential improved, showing a color difference to blue. Identical to the UV–vis spectra effect, there was no substantial distinction within the color coordinate. At the same time, the potential was more elevated than −0.9 V. This behavior is backed through the device's pictures at different potentials (Figure 8f).

A combination of an ABA type copolymers with ILs was shown by Lodge and Ueki^[^ ^76^ ^]^ to form an IGs. The fabricated ion‐gel‐based materials were used by various devices like the casting of solvents, printing with aerosol jet, etc. It was also found that these IGs are used in electroluminescent and electrochromic display with sensors, supercapacitors, etc. They are also a good substitute in biocompatible and biorenewable systems.^[^ ^77^ ^]^ In this an ion gel was prepared by using self‐aggregation of triblock copolymers poly(styrene‐*b*‐ethylene oxide‐*b*‐styrene) and poly(styrene‐*b*‐methyl methacrylate‐*b*‐styrene) with IL (room temperature) 1‐ethyl‐3‐methylimidazolium bis(trifluoromethylsufonyl)imide, the ionic conductivity lies from 3 × 10^−5^ to 3 × 10^−2^ S cm^−1^ and specific conductance 0.3 to 10 µF cm^−2^.^[^ ^78^ ^]^

#### 3.2.3 Classification Based on the Type of Reinforcement

Reinforcement is strong inactive and unwoven fiber‐like materials mixed with matrix to enhance the physical and mechanical properties, e.g., boron, jute whiskers, asbestos, etc. Based on reinforcement, polymer composite is classified as follows.

##### Fiber‐Reinforced Composites

In today's scenario, we need low cost, lightweight materials with high mechanical properties and good chemical resistance. To attain these achievements, fibers or carbon fibers composite is used as reinforcement material that adds desirable properties to composites, e.g., carbon fibers, glass fibers like metal and ceramic used latterly to make composite materials stiff and heat resistant.^[^ ^79^ ^]^

Fibers composite material has three integral parts.

- Discontinuous face as fibers which is also known as the dispersed phase.
- Continuous space as a matrix.
- Interface is also known as the fine interface part.

The effect of fibers composite can be evaluated by length, structure, constituents of fibers, and the mechanical impact of the matrix. The alignment of fibers also plays an essential function in the strength of the composite, i.e., the longitudinal direction has excellent strength. Many organic as well as inorganic fibers are used as strengthening materials in the composite. Organic fibers like glass, silicon carbide, carbon fibers or graphite fibers, some multiphase fibers etc., are used, which are of the high elasticity, high thermal strength, and greater hardness compared to organic fibers. On the other hand, organic fibers with low density are stretchable and bouncy.

##### Laminar Composites

Laminar reinforcement composite is used to enhance the mechanical properties of structural units. Laminated composites are made from stacking various layers, leading to improvements of strength and hardness (like in the case of alumina with zirconia). Laminates are based on tape casting, which allows only a preferred orientation of the reiforcements. Laminar composites tend to be prepared using an organic matrix, leading to the stacking of layers into the different architectures.^[^ ^80^ ^]^ Those are used primarily in electronic ceramics. Polymeric reinforcements and matrices tend to be used quite extensively in transport, aerospace, and constructions applications. Strength and toughness are achieved in laminar ceramic composites by either providing the reinforcement inside the layer, and/or reinforcing the layer interface.

##### Particulate Materials

Particulate reinforced composites contain hard material dispersed uniformly in a matrix. Those composites are used in engineering components because of their mechanical property. Examples are metal matrix and polymer matrix composites.^[^ ^81^ ^]^ The composites show yield, fatigue, and strength, but in general poor ductility, especially in the case of thermoset matrices. From the early stage of the formation, cracking MS of particles or bonding damage at the matrix interface occur. Particulate‐based materials are in general classified as follows.

i) Particle reinforced composites, in which particle and matrix do not interact at the atomic/molecular level. Particles are significantly stiffer than the matrix; hence load bearable, e.g., concrete.
ii) Dispersed strengthened composites, in which particle and matrix interact at the atomic level, leading to strengthening, e.g., thoria dispersed nickel.

The performance of these composites is based on

- particles’ diameter,
- interparticle distance, and
- reinforcement volume fraction.

Besides the above‐discussed reinforcement materials, other components like fillers (i.e., particle‐filled and microspheres), whiskers, flakes, directionally solidified eutectics, etc., are also used to enhance or improve various desirable sets of properties of polymer, metal or ceramic‐based composites.

### 3.3 Classification Based on Ionogel

**Figure 9:**
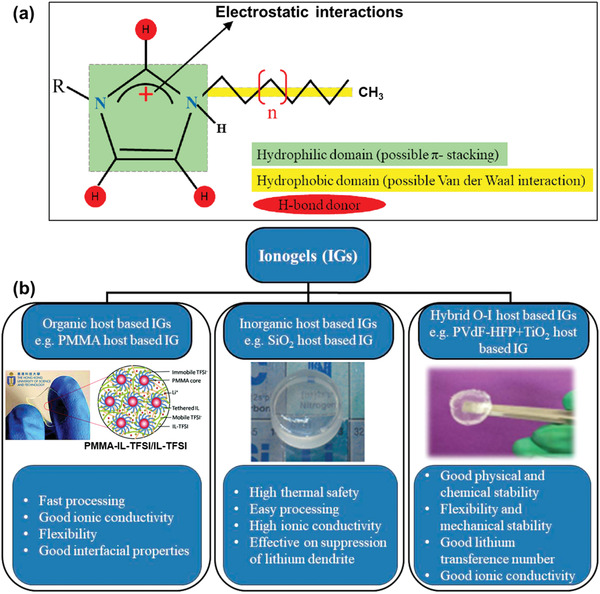
a) Imidazolium based IL shows various interacting sites. b) Dissimilar classes of IGs founded upon host constituents. Reproduced with permission.^[^ ^82^ ^]^ Copyright 2021, Elsevier Ltd.

ILs consist of some distinctive chemical arrangements that can generate a series of attractive forces like van der Waals, electrostatic, Coulombic forces, and hydrogen bonds. ILs can be applied to different applications by tailoring the choice of particular cations and anions. The various interacting sites of an IL cation imidazolium are shown in **Figure** **9**a. Based upon the specific support host of the doped ILs, IGs may be categorized within three distinct types (Figure 9b), i.e.: i) organic (polymers), host‐based IGs, ii) inorganic (oxide grids, oxide nanocomposites, etc.) host‐based IGs, and iii) hybrid organic–inorganic host‐based IGs. In IGs, ILs are generally adsorbed on the solid/porous connected network or integrated/trapped into a polymer system.

In essence, IGs are solid‐electrolyte based ILs and are prepared by the introduction of ILs into solid organic or inorganic type host substances. Compared to conventional ILs, IGs exhibit better chemical, thermal, electrochemical, and mechanical stability and good ionic conductivity.^[^ ^82^ ^]^ During the formation of IGs, ILs act as structural media while the formation of inorganic IGs occurs. IGs carry all the characteristics of ILs except the nature of outflow. On the other hand, like ILs, they also allow shape‐changing and have many applications. As a result, IGs are used to form good types of solid electrolyte membranes used in all types of solid appliances like fuel cells, lithium batteries, dye sensitized solar cells, etc. Further, the function of IGs improved by two methods: i) introduction of organic function in solid matrix, and ii) encapsulation of some molecular moiety into the stable IL phase, this leads to fabrication of some advanced substances like sensors, drug releasing system, formation of biocatalytic membranes, etc.^[^ ^83^ ^]^

Fujii et al.^[^ ^84^ ^]^ also fabricated tetra‐arm poly(ethylene glycol) (Tetra‐PEG) IGs by introducing an ILs based on imidazolium into less concentration, i.e., 3 to 6 wt% of Tetra‐PEG. The prepared Tetra‐PEG IG exhibits superior mechanical characteristics as the compression and stretching test results. This IG is also used in electrochemical devices and the formation of membrane that separate and adsorb CO_2_. Amino acid‐based IL is used to make tough IG membrane used in CO_2_ capture.^[^ ^85^ ^]^ Further epoxy‐amine‐based IG membranes were prepared that facilitate the transport of CO_2_ versus CH_4_ in a dry either humid mixed gas supply situation that is appropriate to the separation of biogas.^[^ ^86^ ^]^ Ueki et al. reported a composite material that was based on an IL, namely, 1‐butyl‐3‐methylimidazolium hexafluorophosphate and a triblock ABA copolymer (A = poly(ethylene oxide) B = thermo‐ and photosensitive random copolymers with *N*‐isopropylacrylamide and A = 4‐phenylazophenyl methacrylate including azobenzene chromophore). The composite material shows transformation from sol to gel on irradiation of UV radiation at 47 °C. The spoiled ABA IG performs a photohealing capability that depends on extreme changes in the flow of IL–polymer composite initiated by illumination of light and results into repairing by filling cracks and cracks was fixed by using gelatin with visible light.^[^ ^87^ ^]^ Further, a triblock copolymer photoreversible gelation in an IL is synthesized by Ueki et al.,^[^ ^88^ ^]^ which shows reversible sol–gel transformation at 53 °C. Based on photodimerization of anthracene, a photohealsble IG was fabricated as a movable dative bond. A Tetra‐PEG and anthracene‐based materials are fabricated and introduced with ILs to form an IG. Further, the photohealing of the IGs was done with the help of the photodimerization process.^[^ ^89^ ^]^ A highly sensitive porous IGs are fabricated in which an IL 1‐ethyl‐3‐methylimidazolium bis(trifluoromethylsulfonyl) imide and a cross‐linked polymer poly(ethyl acrylate‐ran‐styrene‐randivinylbenzene) are added with each other by using in situ cross‐linking polymerization further sugar cubes inverse replication. When these porous IGs are applied over skin, the resulting ionoskins show superior sensitivity and good detection power (≈152.8 kPa^−1^ and 400 kPa, respectively). These ionoskins monitor various human activities like swallowing saliva, elbow bending, locomotion, bending and tapping of fingers, and air blow.^[^ ^90^ ^]^ IGs are used in various electrochemical applications like electrochemical display, smart sensing systems, functional electrochemical energy system like production and its storage.^[^ ^91^ ^]^

**Figure 10:**
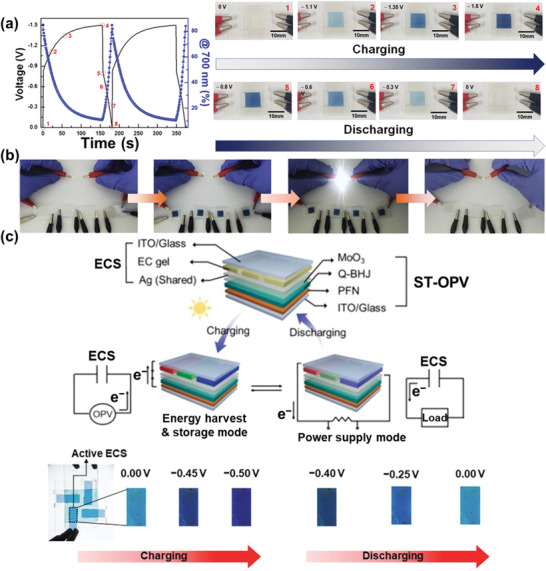
a) Galvanostatic charge–discharge (GCD) curves and in situ transmittance shifts during the CD run and apparatus pictures at eight related matters. b) Process in energy storage and power reserve way of ion gel‐depended ECS. c) Schematic description of the configuration and dual‐mode of monolithically combined OPV and ECS. The charge accumulated in the ECS is shown via a transmittance difference. Reproduced with permission.^[^ ^91^ ^]^ Copyright 2021, American Chemical Society.

The ion gel was deposited upon the WO_3_‐doped ITO probe and then positioned another ITO plate at the canopy of the gel. The ideal EC features of the apparatus contained a high coloration efficiency of ≈61.9 cm^2^ C^−1^, a significant transmittance disparity of ≈91%, and better coloration/bleaching cyclic strength around 10 000 runs. Significantly, the EC behavior was well‐synchronized, including the optical evolution (**Figure** **10**a). The optimized areal capacitance was ≈13.6 mF cm^−2^. The experimental feasibility of the ECS was confirmed, as illustrated in Figure 10b. As the accumulated energy raised through charging, a more robust color intensity was followed. The accumulated energy was reusable for the process of outer electronic segments. For instance, the LED bulb hinged upon successfully while powered through four ultimately charged ECSs in sequence. Currently, an illustrative application of ECSs has been indicated. To provide the dual role of energy conversion and repository and kinds of ECSs and organic photovoltaics (OPVs) were monolithically combined via transmitting one electrode (Figure 10c).

An IG was prepared by Tang et al.^[^ ^92^ ^]^ with the addition of an IL having low toxicity, i.e., 1‐butyl‐1‐methylpyrrolidinium bistrifluoromethanesulfonylimide and a triblock polyester poly(*ε*‐decalactone)‐*b*‐poly(dl‐lactide)‐*b*‐poly(*ε*‐decalactone). A thermoplastic elastomer‐type self‐aggregated cross‐linking is formed, leading to the mechanical enhancement with same processing situations, further hydrolytic breakdown of these IGs possible due to the presence of the ester backbone.

## 4 Applications of ILs‐Based Polymer Composites

**Figure 11:**
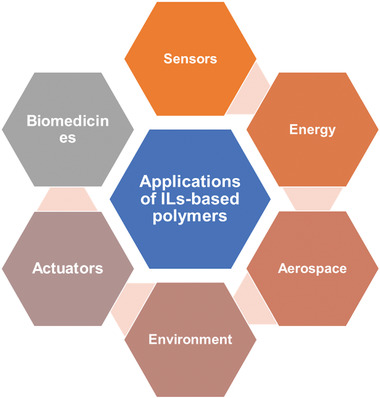
Various applications of ILs‐based polymer composites.

Polymer composites can provide a suitable platform to develop innovative materials with excellent prospective of applications in many fields. Combining a matrix with fiber allows for emerging elegant resources, which harmoniously unite the characters of definite fiber and matrix.^[^ ^93^ ^]^ **Figure** **11** shows the various applications of ILs‐based polymer composites.

### 4.1 ILs‐Based Polymer Nanocomposites for Sensor Applications

Sensing technology comprises many parts, like sensors; another is a data acquisition and transmission system used to verify structural deeds and conduct of structures whenever exposed to circumstances beyond one's control, like an earthquake. The structural health monitoring devices can record actual load, reactions, and forecast environmental measures^[^ ^94^ ^]^ as a sensor polymer composite material used in various fields like medical, agriculture, defence, etc.

#### 4.1.1 Biosensor

Biosensing incorporates an essential function in human beings living for the diagnosis of diseases and examination of diseases. The growth of susceptible, low cost, and selective devices is an excellent way to screen for diseases early. Biosensors are used extensively to recognize biomolecules like dopamine (DA), ascorbic acid (AA), and uric acid.^[^ ^95^ ^]^ Polymer composites based on graphene (Gr) are widely used as a biosensor because of their high mechanical strength, excellent electrical conduction, and biocompatibility.^[^ ^96^ ^]^ Gr‐based polymer composites got much attention for their practical application in enzyme biosensors, DNA biosensors, apt sensors, immunosensors, and optical biosensors.^[^ ^97^ ^]^ It is used for enzymatic biosensors based on the detection of enzymatically generated hydrogen peroxide (H_2_O_2_), electron transfer mediator electrochemistry, and direct electrochemistry of enzyme.

**Figure 12:**
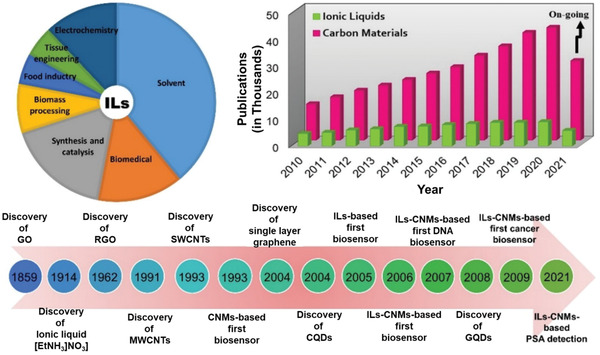
Pie graph of usages of ILs and chronological finding and applications viewpoint of ILs and CNMs. The bar chart shows the numeral of articles every year from 2010 to 2021. Reproduced with permission.^[^ ^98^ ^]^ Copyright 2021, MDPI.

The biocompatibility, comfort of transformation, and preparation of high‐performance nanocomposites of graphene oxide (GO) as well as rGO and easy electrode incorporation are the edges of the GO and rGO into biosensor implementations. **Figure** **12** illustrates the finding and usage of carbon nanomaterials (CNMs) and ILs and their yearly publications, showing that ILs and CNMs are currently becoming researchable fields.^[^ ^98^ ^]^

##### Electrochemical Biosensor

**Figure 13:**
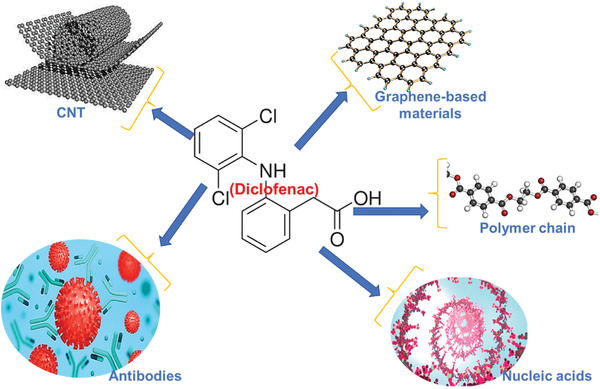
Various materials that are used in electrochemical biosensing of diclofenac.

In today's scenario, we all are dependent on various drugs for our survival. These drugs are chemical substances; sometimes, these drugs have side effects on different body parts when taken in overdoses. For monitoring these drugs, we need some analytical tools. The most suitable option for this is electrochemical biosensing. Boumya et al.^[^ ^99^ ^]^ developed an electrochemical biosensor to detect diclofenac, an analgesic. **Figure** **13** shows various materials that can be used in electrochemical biosensing of diclofenac.

An electrochemical sensor that is simple protein molecularity imprinted is basically based on a chitosan/IL‐Gr customized glassy carbon electrode (GCE); the sensor exhibits superior performance for detecting bovine serum albumin (BSA).^[^ ^100^ ^]^ Electrochemical biosensor based on IL‐based polymer composite is also used to detect microbial pathogens present in food and foodborne pathogens. Nowadays, many works are done on this, so we have also paid attention to the selectivity and sensitivity of ILs based electrochemical biosensors.^[^ ^101^ ^]^ These electrochemical biosensors work very efficiently to detect various biomolecules in our body like enzymes, carbohydrates like glucose, proteins like myoglobin (Myo), hemoglobin (Hb), etc., for the body's proper functioning. Some of them are explained below.

###### Enzyme's Biosensors

**Figure 14:**
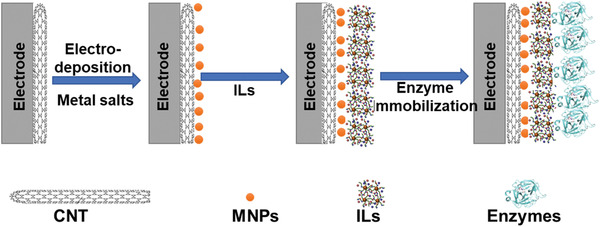
Fabrication of CNT‐MNPs/ILs‐based biosensor electrode.

Enzymes are chemically protein molecules; they work like a catalyst in our bodies, which is why they are also known as biocatalysts. Enzymes control all biochemical activities in our body like digestion, protein synthesis, DNA replication, etc. ILs enhanced the performance of various enzymes for biosensing of various materials—IL–polypyrrole–Au (IL–PPy–Au) composite used for enzyme immobilization for H_2_O_2_ sensing. A new electrochemical biosensor based on enzyme has the benefit of IL–PPy–Au composite to immobilize horseradish peroxidase (HRP) on the GCE. The synthesized C_12_–PPy–Au–HRP/GCE based biosensor has an excellent electrochemical activity for detecting H_2_O_2_.^[^ ^102^ ^]^ An IL‐based polymer composite based on cholesterol oxidase (Chox) enzyme acts as a biosensor. It has an outstanding performance for the cholesterol of a less quantity of 0.5 to 5 mm having excellent sensitivity, less responding time, and more stable repeatability.^[^ ^103^ ^]^ The above‐mentioned IL‐based amperometric biosensor was prepared by using chitosan (Chi)–IL (1‐butyl‐3‐methyl imidazolium tetrafluoroborate) [BMIM][BF_4_],^[^ ^104^ ^]^ which is a cross‐linked matrix, and gold nanoparticles (AuNPs) onto thiol (‐SH) based multiwalled carbon nanotubes (MWCNTs) with cholesterol oxidase (Chox) enzyme. The synthesized biosensor is designated as‐MWCNT(SH)–Au/Chi–IL/Chox. **Figure** **14** shows the fabrication of the CNT‐MNPs/ILs‐based biosensor electrode.

Lu et al.^[^ ^105^ ^]^ prepared an HRP‐based biosensor, which has no reagent and works on the direct transfer of an electron between enzyme and electrode. It shows direct electrochemistry as well as bio‐electrocatalysis of HRP for H_2_O_2_. To prepare this HRP biosensor, the composite material is made up of Chi with room temperature‐based IL [BMIM][BF_4_]. This gives an efficient method for realizing the electrochemistry of various enzymes and exploring third‐generation biosensors.

###### Glucose Biosensors

Glucose (Glu) is a carbohydrate, and they are known as body fuel; in this, Glu comes into the monosaccharide part of carbohydrates. Glu is the most straightforward carbohydrate that our body absorbs directly and uses in the various metabolic process of the human body. Glu breaks down into gluconic acid during respiration and releases a tremendous amount of energy. However, sometimes due to some catalytic activities, it increases body blood sugar levels, leading to significant problems like kidney failure, liver failure, etc., this condition is known as diabetes.^[^ ^106^ ^]^ In this way, various enzymatic and nonenzymatic biosensors developed from time to time. The enzyme‐based sensor, namely, Glu oxidase (GOD), catalyzes the glucose to facilitate the recognition of the Glu amount in the blood.

**Figure 15:**
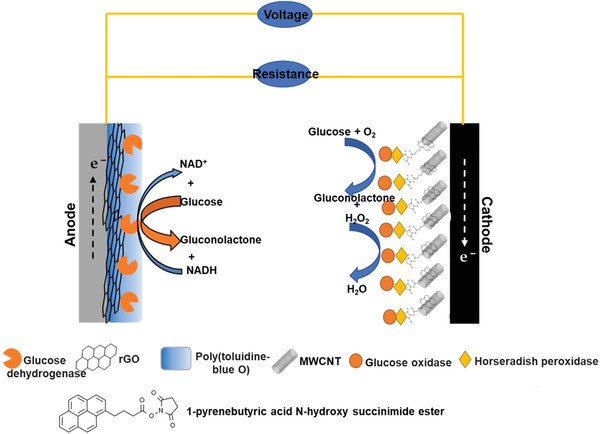
Schematic arrangement of the Glu self‐energy biosensor designed established upon an enzymatic biofuel cell.

On the other hand, in the case of a nonenzymatic sensor, a suitable substance is fabricated on the sensor's surface, which goes with the catalytic activity against glucose to catalyze the Glu^[^ ^99^ ^]^ suitably. A nonenzymatic electrochemical Glu sensor was fabricated that was made up of mixed NPs of Ni–Pd modified IL–rGO on the GCE.^[^ ^107^ ^]^ The detection of Glu using this sensor shows that it had a broad array of linear Glu detection, i.e., from 0.0002 to 10.0 mm and a limit of detection of 0.03 µm and sensitivity of 1504.61 mA mm ^−1^ cm^−2^.^[^ ^108^ ^]^ An enzymatic glucose biosensor was developed based on GOD and HRP enzymes. Further, it is combined with an IL‐based polymer composite. The above‐discussed biosensor was prepared as with which a 3D microporous (3DM) N[3‐(trimethoxy silyl) propyl] aniline (PTMSPA) polymer blended with an IL. [BMIM][BF_4_] deposited on electrode of ITO by electrodeposition method, in which PS spheres were used as a sacrificial template. The 3DMPTMSPA‐IL composite had distinctive characteristics like a huge available surface, better biocompatibility, and outstanding chemical stability, an ideal bioassay. The Glu biosensor was prepared by immobilizing GOD and HRP on the surface of 3DM composite film by using Nafion (Nf) as binding material. The biosensor known as Nf–GOD–HRP/3DM–PTMSPA–IL/ITO has an electrochemical performance for glucose over a large concentration array having excellent selectivity as well as sensitivity. It is used in the detection of Glu in the serum of humans.^[^ ^109^ ^]^ **Figure** **15** shows the arrangement of the Glu self‐energy biosensor designed established upon an enzymatic biofuel cell.

Regarding the Glu biosensors above, the Glu biosensors based on the electron transfer mechanism paid much attention due to the absence of mediators and are referred to as third‐generation biosensors. These IL‐based Glu biosensors are very important to detect blood sugar levels due to their high sensitivity and selectivity.

###### Hemoglobin and Myoglobin

**Figure 16:**
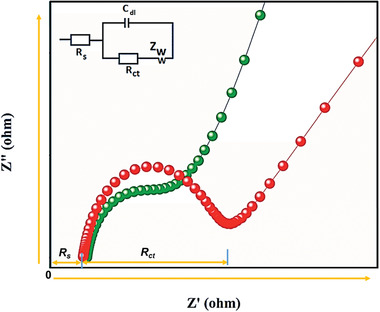
A study overview of EIS, which is being used under electrochemical study.

Hb and Myo both are proteins and bind oxygen to transport it to the body, but the primary difference between them is that Hb is a heterotetrametric protein and present in erythrocytes; on the other hand, Myo is a monomeric protein and found in muscles tissue in which it is intracellular storage site of oxygen. The IL‐based composite resources can be widely utilized as an immobilization matrix to capture proteins and enzymes. Hb was selected as a sample protein to study the hybrid method. A novel composite material, Hb–Chi–BMIMBF_4_/GC, gives a good electrochemical sensor platform for reduction–oxidation proteins and enzymes, and it has various electrochemical applications in biosensors, direct electrochemistry, and biocatalysis.^[^ ^110^ ^]^ A novel electrochemical biosensor was prepared by immobilizing Hb on a DNA customized carbon IL electrode (CILE) using [EMIM][BF_4_] as the modifier. The obtained electrochemical impedance spectroscopy (EIS) peaks on the customized electrode show that the Nf and DNA composite material gives a unique biocompatible microenvironment for maintaining the indigenous construction of Hb and enhancing the rate of direct transfer of Hb by using the basal electrode. The electrochemical variable of Hb in the composite film was additionally considered by using the outcomes of the charge transfer coefficient (*α*) and the apparent heterogeneous electron transfer rate constant (*k*_s_) as 0.41 and 0.31 s^−1^. The prepared electrochemical biosensor exhibited outstanding electrocatalytic performance for reducing trichloroacetic acid (TCA), H_2_O_2_, NO_2_^−^, and the noticeable Michaelis–Menten constant (*K*_M_^app^) for the electrocatalytic response were deliberated, respectively.^[^ ^111^ ^]^ **Figure** **16** shows the study overview of EIS, which is being used under electrochemical study.

A noble electrochemical biosensor was prepared to spot acrylamide and was based on Hb capture in IL‐carbon paste. The Hb–carbon IL paste electrode (Hb/CILPE) was examined using various electrochemical techniques. The planned biosensor shows superior sensitivity and low observation limit for AA with the standard sample preparation method. That is why the Hb/CILPE must be used for direct electrochemical determination of acrylamide in food samples.^[^ ^112^ ^]^

The molecularly imprinted technique is a good substitute for biomolecules recognition systems, including immunoreactions, enzyme‐catalyzed reactions, aptamer, etc. For this, the IL 1‐3‐vinyl imidazole bromide was prepared and used to invent a molecularly imprinted layer for making electrochemical sensing of Myo. This layer was decorated on a GCE modified with MWCNT using the IL as the functional monomer. The sensing behavior of the customized electrode was studied by using the hexacyanoferrate structure as an electrochemical reduction–oxidation probe. The outcomes show that the sensor has better selectivity, excellent sensitivity, competent techniques for medical diagnostics, environment protection, and food safety.^[^ ^113^ ^]^

A unique biosensor that is made up of electro‐co‐deposition of Myo, sodium alginate (SA), Fe_3_O_4_–graphene (Fe_3_O_4_–GR) composite on the CILE was constructed by using Nf as the thin layer making substance to develop the constancy of protein immobilized on the electrode's surface, and the fabricated electrode was represented as Nf/Myo–SA–Fe_3_O_4_–GR/CILE. The invented third‐generation biosensor showed a wide linear range from 1.4 to 119.4 mmol L^−1^, less detection limit as 0.174 mmol L^−1^ (3*σ*), better stability and reproducibility for the reduction of TCA, which would be effective of being a potential biosensing proposal in the electroanalysis and electrocatalysis. Further, a biosensor was prepared for the recognition of H_2_O_2_ that was based on a Myo composite of Nf and a room temperature IL, namely, 1‐butyl‐3‐ methyl‐imidazolium chloride [(BMIM)Cl]. The projected biosensor has an inferior detection boundary as compared to many other biosensors that was based on IL–heme protein and it excluded from general interference in H_2_O_2_ biosensors.^[^ ^114^ ^]^

###### Neurotransmitters

**Figure 17:**
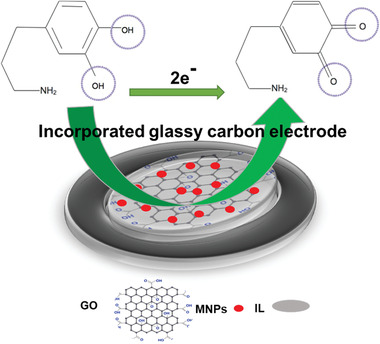
Schematic depiction of the electrochemical sensors for detecting DA on incorporated GCE.

Neurotransmitters (NTs) are endogenous hormones that assume a significant role in various brain functions; an anomalous amount of these NTs causes many bodily, psychotic, and neurodegenerative ailments like Alzheimer's, Parkinson's, and Huntington's disorder. That is why, their sensitive and vigorous recognition is an immense clinical consequence—the electrochemical recognition of NTs done by enzymatic and nonenzymatic methods.^[^ ^115^ ^]^ Epinephrine (EP) is the most essential NTs in mammalians’ central nervous system. Change in EP concentration results in many disorders, so the detection of EP is essential. Unfortunately, EP and AA exist simultaneously in a biological system. The oxidation of AA takes place near the potential of EP at mainly solid electrodes resulting in a coincidental voltametric reaction for EP and AA.^[^ ^116^ ^]^ To overcome this problem, one can use a modified electrode that can selectively detect the EP in the existence of AA. A new rGO composite GCE modified with IL crystal (ILC), 1‐butyl‐1‐methylpiperidinium Hexa‐fluoro‐phosphate [BMPM][PF_6_] and cyclodextrin (CD/ILC/RGO/ILC/GC) was made by the mechanical casting of every layer. The personalized electrode was used for the detection of some NTs like DA, EP, norepinephrine (NEP), levodopa (L‐DOPA), 3,4‐dihydroxy‐phenylacetic acid, and serotonin (ST).^[^ ^117^ ^]^ Atta et al.^[^ ^118^ ^]^ synthesized a new carbon‐composite electrode, i.e., based on ILC [BMPM][PF_6_] and nickel oxide NPs. They were successfully used as an electrochemical sensor to detect paracetamol in a sample of human urine and some NTs like DA, NEP, L‐DOPA, and ST. Further, an electrochemical biosensor that was used to know the presence of DA and generally based on IL decorated GO supported NPs of gold (Au), i.e., (IL–GO–AuNPs) that was coated on a GCE.^[^ ^119^ ^]^ **Figure** **17** shows the schematic illustration of the electrochemical sensors for detecting DA incorporated GCE.

#### 4.1.2 Humidity Sensor

**Figure 18:**
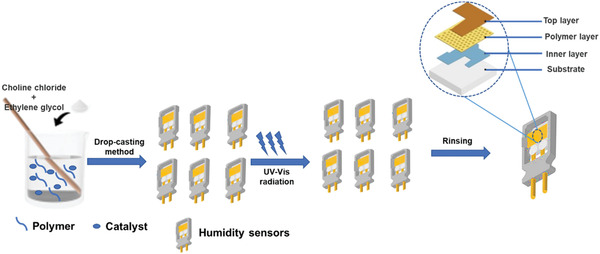
The graphical preparation of humidity sensor and description of the layers.

Humidity is an essential factor for living beings and the environment. The observation of the evolution and role of humidity plays a crucial function in various fields, like medicine, farming and electronics corporations, and health observance. Different components like ceramics, Gr, and polymers promote humidity sensors. Thermoplastic and thermoset‐based composites with natural fibers like flax, hemp, and choir and special stacking sequences also show sensitivity and shape change capabilities based on the level of surrounding relative humidity.^[^ ^120^ ^]^ It is however a significant challenge to design humidity sensors with all the most desirable properties, i.e., brilliant linearity, high sensitivity, small hysteresis, fast response time, and many more.^[^ ^121^ ^]^ Detection of low humidity is always a challenge. In that case, IL ligands were evenly established in a framework of UIO‐66 to enhance the hydrophilic nature of the sensing substances.^[^ ^122^ ^]^ **Figure** **18** shows the preparation of the humidity sensor and a description of the layers.

**Figure 19:**
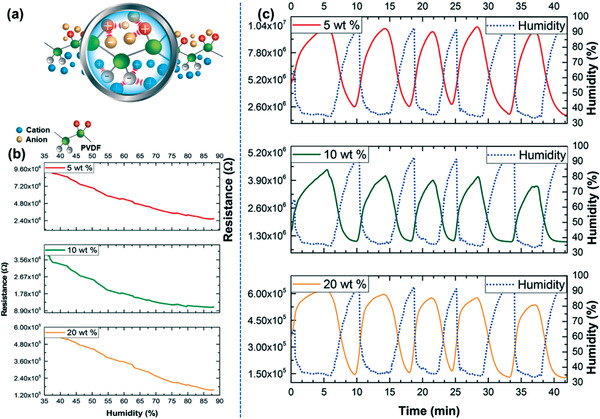
a) A interaction, i.e., ion–dipole in between PVDF and [BMIM][FeCl_4_] in humidity sensing. b) Humidity sensor responsivity to the [BMIM][FeCl_4_]/PVDF with distinct constituents of [BMIM][FeCl_4_]. c) Sensor sensitivity toward fluctuating humidity circumstances to the [BMIM][FeCl_4_]/PVDF through diverse components of [BMIM][FeCl_4_]. Reproduced with permission.^[^ ^123^ ^]^ Copyright 2019, American Chemical Society.

Fernandes et al.^[^ ^123^ ^]^ synthesized a new humidity sensor that was based on polymer composites comprising IL [BMIM][FeCl_4_] and the PVDF. The moisture reaction of the [BMIM][FeCl_4_]/PVDF sensor materials consisting of various IL amounts was analyzed by changing the resistance through changeable relative humidity (RH) from 35 to 90% RH at a steady temperature of 25 °C. **Figure** **19**a shows an interaction, i.e., ion–dipole in between PVDF and [BMIM][FeCl_4_] in humidity sensing is present. The reproducibility of various specimens’ sensors was estimated using [BMIM][FeCl_4_]/PVDF compounds to replicate humidification/dehumidification processes. The outcomes are exhibited in Figure 19b via curve, the function of time is resistance for five humidity processes beneath a humidity deviation from around 35% to 90% RH and consistent heat of 24 °C. The sensor delivers significant constant resistance for the five humidity processes. Figure 19c illustrates the evolution of the resistance, including the humidity; the most elevated resistance values are marked for the lower humidity (35%), the resistance is decreasing with growing humidity accumulation.

#### 4.1.3 Piezoresistive Sensor

In the case of piezoresistive sensors, resistivity is dependent on the strain. Acrylate‐based polymers are used in union with ILs to support the manufacturing of elastic piezoresistive touch‐based sensors. The execution of the piezoresistive sensor was valued up by the degree of the cross‐linking and molecular heaviness of the matrix that controls the ability of movement of the polymeric chains and IL domain. The polymerization and cross‐linking are managed by changing UV exposure time and the amount of cross‐linking agent, respectively, which finally impact the sensitivity of the sensors. It was manifest that the piezoresistive behavior turned into the reorganization of polymeric chains and IL domains within the composite formed through the contortion of the components.^[^ ^124^ ^]^ Piezoresistive strain gauge sensors are the most widely used sensor among all types of strain gauges used to measure acceleration, force, torque, pressure, and vibration. IL/polymer composites (1‐ethyl‐3‐methyl‐imidazolium tetrafluoroborate [EMIM][BF_4_]/2‐[[(butylamine) carbonyl]oxy]ethyl acrylate (BACOEA)) were fabricated to use as sensing substances for stretchable piezoresistive tactile sensors.^[^ ^125^ ^]^ The activity of the piezoresistive sensors was explored with the extent of cross‐linking and polymerization of the IL/polymer composites.^[^ ^126^ ^]^

**Figure 20:**
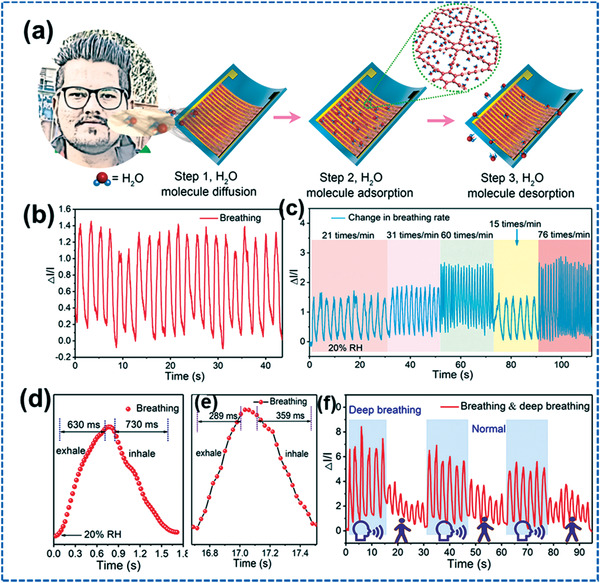
a) Visual illustration of the breathing process. b) Appliance retort to biological breathing. c) Instrument retort to diverse breathing swiftness. d,e) Substantial indication retort to extinction and breath while regular breathing and behind entire movement breathing duration. f) Appliance arrangement under deep breathing occurrences. Reproduced with permission.^[^ ^127^ ^]^ Copyright 2022, Elsevier Ltd.

The complete living sensing process is displayed in **Figure** **20**a. Figure 20b illustrates the response 5 cm from the sensing instrument through the terminus. Besides, these sensors might respond to exhaling on various frequencies within concurrent due to graphdiyne's quick absorption, desorption, and response characteristics to water elements. As expressed in Figure 20c, the breathing rate obtained using the resulting curve is consistent with the identical respiration swiftness, demonstrating the probability of quantifiable examination of respiratory velocity. Here, a distinct reaction toward one breathing duration is depicted in Figure 20d, including the puff humidity growing from 20% to 35%. The puff rate enriched quite following vital moves, abruptly breath, and respiration techniques demonstrated in Figure 20e. Figure 20f illustrates instrument interpretation under profound breathing occurrences.^[^ ^127^ ^]^

**Table** **1** shows the IL‐based biosensing materials and their different applications.

**Table 1:** Ionic liquid‐based biosensing materials and their different applications

Sr. no.	Name of IL	Materials	Applications	Refs.
1.	[BMIM][TfO] (trifluoromethyl sulphonate) + [EMIM][TFSI] (Bis(trifluoromethyl sulphonate) imide)	Propylene carbonate	Ammonia sensing	^[^ ^128^ ^]^
2.	[BMIM][BF_4_]	Chitosan	Protein and enzyme sensing	^[^ ^110a^ ^]^
3.	[VEIm][DCA] (1‐Vinyl‐3‐ethyl imidazolium dicyanamide)	Ammonium persulphate and silica	Self‐relieving sensor for detection of respiration	^[^ ^129^ ^]^
4.	[BMIM][PF_6_]	Poly‐*N*‐succinimidyl acrylate	Glucose sensor	^[^ ^130^ ^]^
5.	[EMIM][BF_4_]	2‐[[(butylamino) carbonyl]oxy]ethyl acrylate	Piezoresistive tactile biosensor	^[^ ^126^ ^]^
6.	[BMIM][FeCl4]	Poly(vinylidene fluoride)	Humidity sensor	^[^ ^123^ ^]^
7.	[BMIM][TFSI]	Propylene carbonate	Ammonia sensor	^[^ ^128^ ^]^

### 4.2 ILs‐Based Polymer Nanocomposites for Energy Related Applications

As the population increases rapidly, the energy demand also increases. A possible material to be used to meet energy management requirements from various alternative energy sources is an ILs‐based polymer composite for broad energy production applications. the integration of ILs to the solid polymer electrolytes could also improve the ionic conductivity, due to superior energy density, broad electrochemical window, thermal stability, minor vapor pressure, and flameproof.^[^ ^131^ ^]^ ILs also used to develop quasi‐solid‐state electrolytes (QSSEs), which affect the activity of quasi‐solid state lithium metal batteries, enhancing the ionic conduction of selected electrolytes with their stability. ILs are also used to prepare high‐responding electrodes because of their distinctive microstructures.^[^ ^132^ ^]^ As we discussed above, IL polymer‐based composites have many applications in the field of batteries like QS lithium metal batteries (QS‐LMBs), lithium‐ion batteries (LIBs),^[^ ^133^ ^]^ lithium‐oxygen batteries,^[^ ^134^ ^]^ as well as in supercapacitors (SCs),^[^ ^135^ ^]^ fuel cells,^[^ ^136^ ^]^ etc.

**Figure 21:**
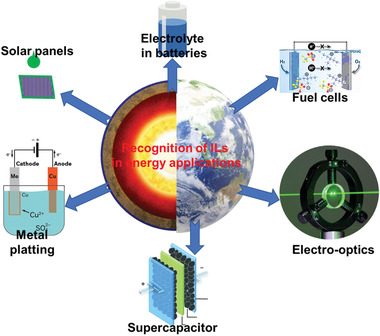
Representation of the recognition of ILs in different energy‐based applications.

The concept of using IG, i.e., IL‐based polymer gel as electrolytes, is a new advance in science that pulls great attention due to their properties and hence applications in energy storage. The combination of ionic liquid and polymer allows to form a solid gel‐like structure that results in superior properties as well as can be used in lithium cell assembly due to their physicochemical properties.^[^ ^137^ ^]^ Besides that, Hernández et al.^[^ ^138^ ^]^ have also reported a synthesis route for poly(ionic) electrolytes that they successfully synthesized via anion exchange resin and explained their broad scope of poly(ionic) electrolytes toward energy storage technologies. Some energy‐related applications of ILs are discussed in this section. **Figure** **21** illustrates representation of the recognition of ILs in different energy‐based applications.

#### 4.2.1 Batteries

ILs are used in batteries due to their safety characteristics in terms of overheating, short circuits, battery explosions, caused by liquid electrolytes. ILs have undergone some significant advances in different areas for the development of battery cells. ILs have also been used to prepare the slurry for producing electrodes, as electrolyte additive in solid electrolyte interphase (SEI), and as a solid polymer electrolyte in various polymer composites.^[^ ^139^ ^]^ Many IL electrolyte‐based batteries are present, like lithium‐based, aluminum‐based, dual‐graphite batteries, etc. We discuss more those below.

##### Lithium Ion‐Based Batteries with Ionic Liquid Electrolyte

There are many Li‐based batteries like LMBs, which are nonrechargeable, known as primary batteries, LIBs and Li–polymer based batteries known as a secondary battery. Li^+^‐based batteries are an essential part of today's scenario due to their daily‐used gadgets like cell phones, watches, computers, tabs, sports equipment, electric vehicles, etc. The coming‐era batteries need the advancement of new ingenious polymers, which is beneficial for better fulfillment on behalf of power density, cyclability, unrefined resources availability, light heaviness, printability, elasticity, and supportability as security.^[^ ^140^ ^]^ High‐thermal stability is expected in IL Li‐based battery. Li electrodes‐based batteries mainly deal with two problems: poor activity and safety problems. So, the characteristics of the junction of two surfaces and the Li–metal surface/electrolyte interactivity are essential to solve the rest problems and improve this appliance. Ternary polymer electrolytes are an excellent option made by poly(ethylene oxide) (PEO), Li salts, and various ILs present in the middle of two Li–metal blocks. The robust cooperation in the electrolytes and the Li‐atoms affects the structure of the electrolyte at its interface area, where minute and flat ions are present in a sound described area along with Li^+^‐ions and with many self‐diffusion coefficients. On the other hand, large ions like (methyloxymethyl) triethyl phosphonium [P222mom]^+^ enhance the density of PEO on the inner side because of the more significant number of species present at the interface. So, the structure‐dynamic characteristics at the interface of electrolyte and Li–metal surface can be improved by selecting particular ILs in ternary polymer electrolytes, which prevent the development of SEI above the exterior part of the electrode in this way enhance the activity of the battery.^[^ ^141^ ^]^

Thermal management is also the main problem in the growing use of batteries. Due to this safety concern, extremely thermally conductive (TC) substances are required. Carbon nanotube (CNT) or fibers filled PVDF having superior TC is the best alternative to traditional electrode substances that carry PVDF and lampblack in Li^+^ cells. Just only adding 8 wt% of graphene nanoflakes, the TC of nanofiber separators of polyacrylonitrile can be enhanced by 3.5 to 8.5 W (m K)^−1^.^[^ ^142^ ^]^ Li‐ion batteries with liquid electrolytes have limitations like low power and energy density, safety, etc. To overcome these problems, solid electrolytes develop, i.e., QSSEs and all‐solid‐state electrolytes. Lately, ILs as a biodegradable substance have paid much concern to emerging QSSEs because of their outstanding properties, like thermally and chemically stable, integrated configuration over an extended variety of working temperatures, more electrochemical stability window, better ionic conduction over normal room temperature with incombustibility. The prepared ILs is categorized as a conventional, solvate, and renewable ILs which is completely bio‐based for the LMBs with QSSEs. The traditional ILs are broadly based on imidazolium, quaternary ammonium, pyrrolidinium, piperidinium, and many more.^[^ ^132^ ^]^

Some scientists synthesized a novel polymerizable IL monomer, i.e., as a repeating unit, linking a meth acryl functional group with the positively charged ion in an IL molecule with comparatively revoked potential windows. A polymer‐based electrolyte is obtained by reacting a small polymer with a binary Li‐IL. The obtained electrolyte shows an outstanding rate discharge property for a Li‐based polymeric battery; it retains 83% of its discharge capacity and comparatively superior cycle activity. This new Li–polymer cell is fireproof and free from leakage, user friendly, big size lithium secondary, i.e., rechargeable battery.^[^ ^143^ ^]^

##### Aluminum‐Based Batteries with ILs‐Based Electrolyte

Reviews of the literature have shown that imidazolium salts or amide ligands (such as urea) can make IL electrolytes or quasi‐IL electrolytes for secondary aluminum batteries. Batteries formed on aluminum provide an applicable option due to their three‐electron redox potential, constancy in the metallic form, and much natural occurrence. The advances of this type of battery are based on fireproof electrolytes with less noxious is essential for reducing safety dangers and environmental effects. Due to this motive, ILs have been proposed for energy‐storing because of their little vapor pressure and more oversized electrochemical windows. A new type of ILs, known as IL analogues or so‐called DESs, usually produced by a mixture of a strong Lewis acid metal halide and Lewis's base ligands, have obtained much consideration due to its own equivalent electrochemical and physical characteristics with less expenditure and lower ecological footmark.^[^ ^144^ ^]^ A 1:1.5 1,2‐ dimethyl‐3‐propylimidazolium chloride:AlCl_3_ electrolyte is used for new rechargeable cell with positive graphite electrode and aluminum as negative electrode works as reversible chlorine (Cl) intercalation electrode. So, separating the membrane between anodic and cathodic electrolytes is not required. 1.7 V average discharge voltage was produced for 1–10 mA g^−1^ graphite, and more than 150 cycles were obtained at the positive electrode at 100% depth of discharge.^[^ ^145^ ^]^

##### Dual Graphite Batteries with IL‐Based Electrolyte

In previous years, much focus has been devoted to the development of cation intercalation‐based batteries, but anions had no consideration or less consideration in the electrolyte. Anion intercalation was achieved through a highly concentrated acid solution as an electrolyte, but significant security concerns made this implementation less valuable. The double graphite interpolates melted electrolyte‐based batteries that gain attention on implementing anion interject graphite as the positively charged electrode in batteries when we used IL as electrolyte at room temperature.^[^ ^146^ ^]^ A battery that shows high reversibility and capacity detention and brings a capacity is a new aluminum–graphite dual‐ion battery (AGDIB) in an electrolyte, i.e., ethyl‐methyl carbonate, having superior energy density.

**Figure 22:**
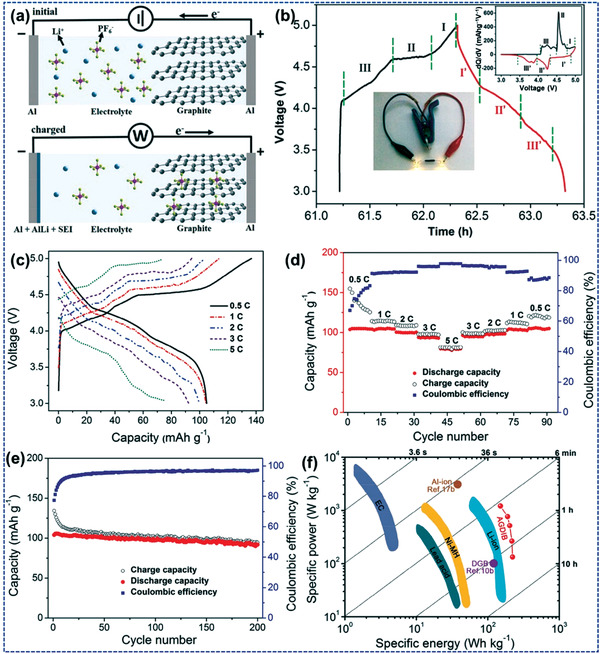
a) Diagrammatic description of the AGDIB in the primary condition (up) and the charged form (down). b) GCD arc of the AGDIB at 0.5 C. Insets are the battery's d*Q*/d*V* differential curve and a picture illustrating a single AGDIB cell igniting up two yellow LEDs within a sequence. c) CD arcs of the AGDIB beneath different current rates. d) Rate capacities and compare coulombic efficiencies of the AGDIB. e) The stability cycle trial outcome of the AGDIB at a current speed of 2 C. f) Execution comparison of the AGDIB with traditional ESS appliances and different newly documented progressive energy storage apparatuses, while DGB defines dual‐graphite battery. Reproduced with permission.^[^ ^147^ ^]^ Copyright 2019, Wiley‐VCH.

**Figure** **22**a diagrammatically shows the early and charged conditions of the AGDIB. At charging, PF_6_^−^ anions within the electrolyte interpolated in the cathode made from graphite. The Li^+^ ions in the electrolyte deposition upon the aluminum counter probe to construct an Al–Li alloy. Figure 22b illustrates galvanostatic charge–discharge (GCD) arcs of the AGDIB, showing a specific anion intercalation/deintercalation shape within graphite. The battery's usual galvanostatic charts are displayed in Figure 22c. Figure 22d additionally exhibits the CD capabilities and approximating coulombic competence of the AGDIB while the rate capability trials. Figure 22e represents the stability cycling interpretation of the AGDIB on a current velocity of 2 C. Figure 22f displays an assessment of the AGDIB with different leading electrochemical energy storage (ESS) machinery.^[^ ^147^ ^]^

Natural graphite electrodes are studded and compared with a synthetic graphite electrode (KS6) via CD test. It was found that a dual‐natural graphite battery shows better performance than a dual‐KS6‐graphite battery, e.g., having superior discharge plateau and best plateau retention. The dual‐graphite system's principle is known by doing a cyclic voltammetry (CV) test. An invented dual‐graphite battery (DGB) formed from the pure [EMIM][TfO] IL electrolyte having a charge/discharge voltage between 1.0 and 3.9 V. The outcomes show that the EMIM^+^ cations exhibit much stable intercalation/deintercalation property as compared to the TfO^−^ anion. The main benefit of the [EMIM][TfO]–DGB includes the well‐being measurement of the IL electrolyte and the plentiful electrode resources. Moreover, the [EMIM][TfO] IL contains only C, H, O, N, F, and S atoms; this IL is an excellent and promising material for upcoming electrochemical energy storage devices.^[^ ^148^ ^]^

Fan et al.^[^ ^149^ ^]^ present new work with Zn/graphite DIB, in which naturally obtained graphite acts as a cathode and zinc metal is used as anode with zinc trifluoro methane sulphonate (Zn(TSO)_2_) 98%, an ionic liquid‐based electrolyte. Zn(TfO)_2_/[EMIM][TfO] electrolyte has a vast electrochemical window of 2.8 V (vs Zn^2+^/Zn) compared to the water‐based systems, and it is also with high ionic conductivity of 7.3 ms cm^−1^. The IL electrolyte can repress the development of dendrite on the surface of Zn. On the other hand, some additional advanced characteristics of the IL electrolyte are extra attention‐seeking, like less volatile nature, incombustible, and elevated temperature constancy. The battery system became safer, between the range of voltage 0.8 and 2.8 V. Placke et al.^[^ ^150^ ^]^ present dual‐ion systems made up of the bis(trifluoromethanesulphonyl) imide (TFSI^−^) anion intercalation to the graphite from an IL electrolyte, named as Pyr_14_TFSI. As an anode material, each metallic Li or Li titanate (Li_4_Ti_5_O_12_) was used; both the materials show excellent compatibility with the electrolyte providing a better potential range.

##### Sodium‐Ion Batteries

Energy alteration and its storage have become the main concern for our benefit in day‐to‐day life. The much suitable form of energy storage in terms of energy density is chemical energy. For this idea, batteries are suitable devices that store chemical energy to give electricity with high conversing potential and no pollutant in gaseous form. For this, much attention is cheaper, secure, rechargeable batteries with enough voltage, capability, and rate potential. Among a range of existing energy storing technologies, the Li‐ion battery, which has dominated the portable electronic marketplace, has become the preferred option to provide energy to the next generation of electric gadgets or appliances and connect electric vehicles. Na^+^ based batteries are a better alternative in place of Li^+^ batteries for fewer expenditure applications due to the less cost and plenty of sodium with comparable intercalation chemistry with Li.^[^ ^151^ ^]^ A study reported composite gel polymer electrolytes (GPEs), which act as electrolyte/separators in sodium ion‐based batteries. The GPEs consist of 0.5 m solution of the sodium trifluoromethanesulfonate (Na‐triflate or NaTf) in IL [EMIM][TfO] enclosed in PVDF‐cohexafluoropropylene) (PVDF‐HFP) scattered with inactive filler aluminum oxide (Al_2_O_3_) and active filler sodium aluminate (NaAlO_2_) molecules. The freestanding layers of the composite GPEs, synthesized from a technique, namely, the solution‐cast method, provide the most favorable ionic conductivity (IC) at room temperature (6.3–6.8 × 10^−3^ S cm^−1^ and 5.5–6.5 × 10^−3^ S cm^−1^ for Al_2_O_3_‐ and NaAlO_2_‐scattered GPEs, respectively), having adequate electrochemical stability and outstanding firmness for temperature up to 340 °C. This property makes this composite NaTf/[EMIM][TfO]/PVDF–HFP–GPEs an attractive electrolyte for Na^+^ batteries.^[^ ^152^ ^]^ Because of its high potential and lower cost, sodium‐ion batteries based on quinone electrodes are more prevalent energy storage devices. However, the main disadvantage of this device is that it has a poor life cycle and less using energy because the quinone electrode dissolved in the aprotic solvents. The forbidden impact of ILs on quinone solvation relates to its tendency of polarization, donor number, and dealings power, as exposed by combined density functional theory and several studies in spectroscopy.

**Figure 23:**
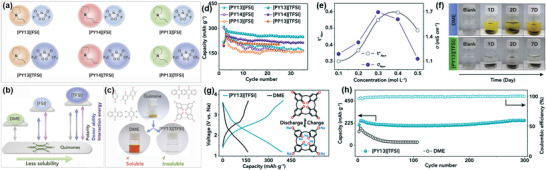
Networks and characteristics of ILs. a) Chemical arrangements of six ILs. b) Graphic assessment of polarity, donor capability, and quinone‐solvent communicating energy for DME and ILs. c) Pictures of soluble quinone within DME and undissolved quinone into IL. Electrochemical features and Na/C4Q Cell implementation: d) cycling interpretation of C4Q cathode on 90 mA g^−1^ (0.2 C) within six IL‐dependent electrolytes. e) Ostensible transport quantity of Na^+^ (*t* ^+^) and Na^+^ ionic conduction (*σ*_Na+_) of [PY13][TFSI]‐dependent electrolyte comprising diverse absorptions of Na[TFSI]. f) Imagining disbanding examinations of C4Q within DME and [PY13][TFSI]. g) The CD arcs of C4Q cathode on 20 mA g^−1^ (0.04 C) into 0.3 m Na[TFSI]/DME and 0.3 m Na[TFSI]/[PY13][TFSI] solution. h) Cycling constancy of C4Q cathode on 130 mA g^−1^ (0.29 C) into 0.3 m Na[TFSI]/DME and 0.3 m Na[TFSI]/[PY13][TFSI] solution. The cathode contains of 50 wt% C4Q, 40 wt% conducting carbon, and 10 wt% binder. Reproduced with permission.^[^ ^153^ ^]^ Copyright 2019, Elsevier Ltd.

**Figure** **23**a illustrates the chemical configurations of the six studied ILs comprised of three cations (i.e., [PY_13_], [PY_14_], and [PP_13_]) and two anions, namely, [FSI] and [TFSI] that were chosen because of their chemical resilience through metallic Na anode. More fragile polarity and more insufficient donor numbers of ILs favors quashed quinone dissolubility (Figure 23b,c). As demonstrated in Figure 23d, the C4Q electrode produces more increased reversible capability into [TFSI]‐based ILs compared to in the [FSI] sequence through the identical cation. With growing Na[TFSI] engagement, both *t* ^+^ and *σ* grow first and decline afterward (Figure 23e). While in Figure 23f, there is no noticeable color difference of [PY13][TFSI] electrolyte for seven days, where the DME electrolyte fast revolves yellow, they are leading the concealed the C4Q solubility in [PY13][TFSI]. Sequentially, volumetric capability and energy density emanated by the critical area beneath the discharge arc are 240 mAh cm^−3^ and 863 Wh kg^−1^_cathode_ (Figure 23g). Even more stirringly, high‐capacity retaining of ≈99.7% and Coulombic efficacy of ≈100% is constant behind schedule 300 runs on 130 mA g^−1^ (0.29 C) (Figure 23h).^[^ ^153^ ^]^

There are many problems in dealing with liquid organic electrolytes. Moreover, the electrolytes present in solid‐state have a much‐established electrochemical window and high protection, which allows highly efficient materials for cathodes and Na metal to act as an anode. Several works have shown that the density of cell energy increases even more than 500 Wh kg^−1^ by using solid‐state electrolytes.^[^ ^154^ ^]^ Furthermore, more activities have taken place in the development of solid electrolyte for Na+ and Li+ batteries for high safety purposes. A hierarchical poly(IL)‐based solid electrolyte (HPILSE) is synthesized for higher, protection in Li^+^Na^+^ batteries. This mixed solid electrolyte is prepared by using in situ polymerization of 1,4‐bis[3‐(2 acryloyloxyethyl)imidazolium‐1‐yl]butane bis[bis(trifluoromethanesulfonyl)imide] (C1‐4TFSI) monomer with 1‐ethyl‐3‐methylimidazolium bis(trifluoromethanesulfonyl)imide‐based electrolyte that is present in poly(diallyl dimethylammonium) bis(trifluoromethanesulfonyl)imide spongy layer. The elegant concordant organization concurrently gives the fabricated HPILSE having better ionic conductivity (>10^−3^ S cm^−1^ at 25 °C) that satisfies electrochemical stability, innate incombustibility, better mechanical potency, and elasticity. The in situ prepared Na_0.9_[Cu_0.22_Fe_0.30_Mn_0.48_] O_2_/Na cells with HPILSE have advanced cycle activity with higher specific capacities. The brilliant act of HPILSE and the easy preparation procedure of HPILSE‐based solid‐state cells make it potentially higher capable electrolyte substances for the future creation of Na^+^ batteries.^[^ ^155^ ^]^

The reported studies in this section show the promise of ILs electrolytes as materials platform for different classes of batteries (lithium ion, aluminum, dual graphite, and sodium‐ion). This section has also discussed the importance and performance of ILs electrolytes in battery applications. Moreover, this section has also provided an overview of the IL‐based materials needed in future energy sector applications.

#### 4.2.2 Fuel Cell

Fuel cell (FC) is a tool that changes fuel's chemical energy into electrical energy without leaving any harmful impact on the environment.^[^ ^156^ ^]^ FCs are usually designed for various working temperatures and the type of electrolyte used, as described in **Table** **2**.^[^ ^157^ ^]^

**Table 2:** Various types and properties of FCs. Reproduced with permission.^[^ ^157^ ^]^ Copyright 2019, Elsevier

Sr. no.	Name of fuel cell	Working temperature	Electrolyte used	Working ability	Output of system
1.	Alkaline fuel cell	90–100 °C	Matrix soaked 60% aqueous solution of KOH	60%	10–100 kW
2.	Proton exchange membrane fuel cell	60–80 °C	Perfluoro sulfonic acid	60:35% for purpose of transport and stationary	1–100 kW
3.	Molten carbonate fuel cell	600–700 °C	Matrix soaked Na_2_CO_3_/liquid solution of sodium lithium	50%	300 kW–3 MW
4.	Solid oxide fuel cell	700–1000 °C	Zirconia stabilized by yttria	60%	1 kW–2 MW
5.	Phosphoric acid fuel cell	150–200 °C	Matrix soaked liquid H_3_PO_4_	40%	400 kW

FCs containing polymer electrolyte membrane have various merits, like more power density, rapid start, less price, and good durability. The development of composites with ILs enhances fuel cell properties and can implant these types of technologies.^[^ ^158^ ^]^ By increasing the content of ILs, thermal stability and the OH— conductivity of the composites increase. Many novel composite membranes were synthesized in laboratory by use of imidazolium‐based aprotic ILs with a polymer matrix, namely, sulfonated poly(ether ketone) (SPEK) with the use of solution casting method due to which the thermal stability (due to electrostatic force of attraction in imidazolium cations of ILs and sulfonic acid anionic groups of SPEK), mechanical characteristics, ion exchange potential, proton's conductance, and leaching out of ILs of a fuel cell increases with water. The membranes based on ionic liquid were more flexible than the pure SPEK film because of the plasticization of the ILs. It was observed that at elevated temperatures, ILs work as the ion charge carrier. Composite membranes of hydrophilic type IL were mechanically stable compared to membranes of hydrophobic type IL.^[^ ^159^ ^]^ Polymer ILs with the IL are used as electrolytes, an ionic conductive membrane, and a catalyst in fuel cell technology with improved properties.^[^ ^160^ ^]^ PILs like *N*,*N*‐diethyl‐*N*‐methyl‐3‐sulphopropan‐1‐ammonium hydrogen sulphate, *N*,*N*‐diethyl‐3‐sulphopropan‐1‐ammonium triflate([DESPA][TfO]), etc., are good electrolytes for FCs working in the temperature array 100–120 °C.^[^ ^161^ ^]^ The combination of two PILs, like diethylmethyl ammonium hydrogen sulphate ([dema]HSO_4_) and diethylmethyl ammonium bis(trifluoromethanesulphonyl)amide ([dema][NTf_2_]) was formed by blending in different weight ratios used as an electrolyte for FC. This mixture results in good electrochemical action compared with the pure PILs, hence the work procedure and speed of action of the mixture depend upon the composition of the mixtures.^[^ ^162^ ^]^

As per the reported studies in this section, the different possible fuel cells and properties also discussed the additional documented material's importance and performance in FCs applications. Table 2 summarizes the various types and properties of FCs.

#### 4.2.3 Supercapacitors

Supercapacitors are electrochemical devices that store and emit energy by the alternative process of adsorption and removal of ions at the interfaces of two surfaces of electrodes and electrolytes. SCs are recent energy‐saving and conversion devices that have the potential of higher power density, excellent circulation properties, fast discharge–charge, less expensive, poor self‐discharging, and safely working mechanism.^[^ ^163^ ^]^ To enhance the performance of energy‐storing devices like an SC, ILs are used as electrolytes. ILs overcome various disadvantages of previously used organic or aqueous electrolytes. ILs are pure liquid salts with a much‐ionized atmosphere, less volatile, wide liquid temperature range, and wide working voltage window. There are many problems with the salvation of ions in bulk in SC. To avoid this problem, ILs are used in electrical double‐layer capacitors (EDLCs), e.g., 1‐ethyl‐3‐methylimidazolium bis(trifluoromethyl sulphonyl)imide [EMZM][TFSI] was used to stay away from the solvation effect.^[^ ^164^ ^]^ On the other hand, when we do the electrolyte's redox decomposition at the carbon electrode, the cell potential of EDLCs decreases; so, the use of ILs with a more electrochemical stability window like substituted pyrrolidinium cations permit the cell voltages more than 3.5 V and the specific energies of commercial EDLCs increases. The asymmetric EDLCs having much specific gravity and some mesoporous positively and negatively charged carbon electrodes of different weights had revealed the growing utilization of pyrrolidinium‐based ILs.^[^ ^165^ ^]^ Tu et al.^[^ ^166^ ^]^ prepared a new redox‐active gel polymer‐based electrolyte with two function IL for flexible supercapacitor in which they chose an IL, namely, 1‐butyl‐3‐methylimidazolium iodide (BMIMI) as a substance that promotes flexibility and reduces the brittleness and redox additive as well as a neutral lithium sulphate (Li_2_SO_4_) solution in water to attain more working voltage. A reduction and oxidation‐active poly(vinyl alcohol)–Li_2_SO_4_–BMIMI gel polymer electrolyte was also designed, and it is used for making a supercapacitor that is flexible and mainly based on carbon. This unique method gives a suitable and less expensive method for efficient energy storing devices. Recently, Lian et al.^[^ ^167^ ^]^ explored the relationship between pore size and adsorption of the ion at room temperature IL capacitor concerning nanoporous electrodes, especially given to attention on capacitance and energy‐storing optimization. Also, the ILs based polymeric gels, also known as “IG,” provide another addition to their use in the energy storage sector. In this regard, the use of EMIMBF4 IL as an electrolyte for supercapacitor applications could be noticed that enables the wide potential window and ILs potentiality toward energy storage applications both with MXenes and quantum dot‐based electrode materials.^[^ ^168^ ^]^

#### 4.2.4 Oxygen Evolution Reaction

The need for sustainable pollution‐free energy sources is critical, and it has developed into one of the most intense technological challenges of the 21st century. H_2_ is an ideal alternative to fossil fuel as it is easy to store and clean fuel for future generations. At present, hydrogen is abundantly produced by the electrolysis of water. Still, it needs voltages in extensive of the thermodynamic water splitting potential of 1.23 V, first and foremost due to the time‐consuming rate of the oxygen evolution reaction (OER). So, some competent catalyst is required to enhance OER's rate and lower the overpotential.^[^ ^169^ ^]^ The utilization of polymer brushes based on IL and poly(IL) was invented by Pham Truong et al.^[^ ^170^ ^]^ as a rising catalyst for oxygen reduction reaction (ORR). The outcome illustrates that the poly(IL) has a competent electrocatalytic performance associated with the chemical constitution and the nanostructuration of the polymer. On the other hand, they also reveal that poly(IL) is also used at the same time like a host–guest strategy for Pt catalyst. For the mixed substances, a capable catalytic importance is for bearance to crossover response. They also disclose the probable uses of poly(IL) and the mixed substances, poly(IL)/Pt, as a proficient dual functional catalyst for ORR and OER.

In this section, the literature reported on OER discussed the IL‐based material's catalytic performance and applications. Also, it examined the additional said IL‐based polymer materials and their multifunctional applications.

#### 4.2.5 Hydrogen Evolution Reaction

In recent years electrochemical splitting of water has taken much interest. An electrocatalyst must be developed with high stability and low potential for effective hydrogen evolution reaction (HER) to implement the successful water‐splitting technology. Mainly, Pt is used as an electrocatalyst for HER, but its high cost is unsuccessful. On passage of time, various transition metals (Co, Ni, Fe) doped with Pt to make low‐cost catalyst, but the problem is also due to corrosion and oxidation of metals in acidic electrolysis. Further, some electrocatalysts are synthesized by doping some heteroatoms like N, P, B, and S with carbon‐based materials for energy‐related electrocatalytic reactions.^[^ ^30^, ^171^ ^]^ In this manner, dicyanamide‐based ILs are used as starting materials to synthesize nitrogen‐doped mesoporous carbons (NMCs).^[^ ^172^ ^]^

The synthesized NMCs had much stability and more reactivity as electrocatalysts for HER. An electrode catalyzed synthesized for HER, which is made up of phosphonium‐based IL [trihexyl(tetradecyl)phosphonium tetrachlorocobaltate(II)], contains cobalt ion, was helpful as new phosphorus and metal dual‐root for preparation of cobalt phosphide. Trihexyl(tetradecyl)phosphonium tetrachlorocobaltate (II) ([P_6,6,6,14_]_2_[CoCl_4_]) with CNTs was used to get the Co_2_P/CNTs as electrocatalyst by single‐step phosphatization. This material shows a good catalytic performance for HER with start overpotential of 85 mV, a Tafel slope of 47 mV dec^−1^, and current densities of 10 and 20 mA cm^−2^ at overpotentials of 150 and 178 mV, and superior stability to maintain the HER performance.^[^ ^173^ ^]^ Further in this manner, Cui et al.^[^ ^174^ ^]^ also prepared IL [*N,N*‐bis(4‐(methoxycarbonyl)benzyl)‐*N*‐methyl‐d‐glucaminium bromide (MBMG‐Br)] having iron (Fe) was added with CNTs as precursors to synthesized CNTs–decorated iron phosphide (FeP_(MBMG)_/CNTs) electrocatalyst for HER. On the other hand, to accelerate the HER, the electrochemical settling of Pt and Pd is done on polymeric brush IL‐based on imidazolium [poly(1‐allyl‐3‐methylimidazolium)]. As a result, various hybrid electrode catalysts have been synthesized. Due to this, the morphology of the nanomaterials changes, resulting in an increased catalytic activity.^[^ ^175^ ^]^

The literature reported in this section upon HER illustrates the IL‐based material's catalytic performance and applications. Also, it discussed the different, said materials fabrication processes and their multifunctional applications.

#### 4.2.6 Oxygen Reduction Reaction

As the need for energy increases day by day, we aim at using energy‐producing appliances that provide energy with high efficiency and less pollution. In this regard, direct methanol fuel cells are the best‐provided option, which come under the proton exchange fuel cell category. In this cell, Pt is abundantly used as a cathode catalyst for ORR, but it has two major disadvantages, one is high cost of Pt, and another is fuel crossover by polymer membrane from anode to cathode. This crossover reduces the potential of cathode and efficiency of fuel. To overcome this problem cathode catalyst synthesized for ORR. In this regard an IL 7‐methyl‐1,5,7‐triazabicyclo [4.4.0] dec‐5‐ene (MTBD) and lithium salt of bis(trifluoromethylsulfonyl)imide (bTFSI), i.e., [MTBD][bTFSI] having much oxygen solubility, have been utilized to manufacture an interior oxygen‐loving electrode, which is nanoporous in nature to improve the ORR activity. On the other hand, by incorporating Gr or IL to Pt catalysts, significant progress has been obtained exclusively in the ORR performance.^[^ ^176^ ^]^ Snyder et al.^[^ ^177^ ^]^ synthesized a composite‐based electrocatalyst for ORR made up of nonporous(np)‐metal‐IL polymer composite, i.e., np‐NiPt infuse with a water hating, protic IL with more O_2_ solubility that shows significant performance in favor of the ORR as comparison to another catalyst that used in this reaction. A superficial and eco‐friendly technique has been invented for the fabrication of much efficient N‐doped Gr by calcination of a regular fusion of GO and conventional water‐loving IL 1‐butyl‐3‐methylimidazolium tetrafluoroborate [BMIM]BH_4_], which act as both N providing as well as a restacking protectant. The catalyst was synthesized at a temperature of 900 °C with a mixing N of about 6.6 at% shows the outstanding catalytic activity for ORR in an alkaline medium.^[^ ^178^ ^]^ Further, an electrode catalyst was synthesized using Pt, Vulcan XC‐72 carbon, 1‐butyl‐3‐methylimidazolium chloride [BMIM][Cl]IL, i.e., Pt@[BMIM][Cl]/XC‐72 composites by an easy precipitation reduction technique. In comparison to Pt/XC‐72 composites, Pt@[BMIM][Cl]/XC‐72 composites show better enhanced electrocatalytic performance and methyl alcohol tolerance toward the ORR in the acidic condition that can be chiefly attributed to electronic factor and steric factor. The competent Pt‐based ORR catalyst shows outstanding electrocatalytic performance and methanol tolerance.^[^ ^179^ ^]^

**Figure 24:**
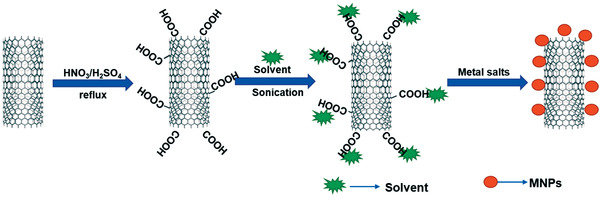
Representation of MWCNT/IL/MNPs hybrid composite formation.

Wang et al.^[^ ^180^ ^]^ fabricated MWCNT/IL/Au hybrid electrocatalyst using a chemical reduction pathway. This proposal implies working MWCNTs with NH_2_‐IL and in sedentary reduction of HAuCl_4_ with no further reductant added to it. The synthesized hybrid composite exhibited excellent electrocatalysis for the reduction of ORR. Additionally, the outcome of experiments proved that both NH_2_‐IL and Au extensively enhance the electrocatalytic performance of MWCNTs. **Figure** **24** displays the graphical representation of MWCNT/IL/MNPs hybrid composite formation.

Some arrays of MWCNT–IL were arranged on silicon (Si) wafers (Si–MWCNT–IL) via a common chemical pathway. The cyclic voltammetry (CV) measurements showed outstanding electrocatalytic performance of Si–MWCNT–IL for ORR in an aqueous medium compared to MWCNT–IL.^[^ ^181^ ^]^

Moreover the ORR also enhanced by engineering the electronic arrangements of Ba_0.5_Sr_0.5_Co_0.8_Fe_0.2_O_3−*δ*_ perovskite oxide with the surface amendment of 1‐butylimidazolium‐3‐N‐propane sulfonate‐*N*,*N*‐bis(trifluoromethyl sulphonyl)amine (BIM3S‐HTFSA) IL.^[^ ^182^ ^]^ **Table** **3** shows the ILs‐based polymer composite in energy storage devices.

**Table 3:** ILs‐based polymer composite in energy storage devices

Sr. no.	ILs	Polymers	Temperature	Applications	Refs.
1.	*N*,*N*,*N*‐trimethyl‐*N*‐(2‐hydroxyethyl)ammonium bis(trifluoromethylsulfonyl)imide [N_1112_OH][TFSI]	Poly(vinylidene fluoride‐trifluoroethylene) P(VDF‐TrFE)	25–110 °C	Solid polymer electrolyte	^[^ ^183^ ^]^
2.	*N*‐*n*‐butyl‐*N*‐methyl pyrrolidinium bis(trifluoromethanesulfonyl)imide [Pyr_14_][TFSI], lithium bis(trifluoromethanesulfonyl)imide [Li][TFSI]	Polyethylene oxide (PEO)	60–80 °C	Li‐ion batteries	^[^ ^184^ ^]^
3.	[Li][TFSI], 1‐butyl‐3‐methylimidazolium bis(trifluoromethanesulfonylimide)[BMIM][TFSI]	Polyamides (PI)	25 °C	Li ion batteries	^[^ ^185^ ^]^
4.	1‐Butyl‐3‐methylimidazolium trifluoromethanesulfonate [BMIMTf]	Hydroxypropylmethyl cellulose (HPMC)	Ambient condition	Solid polymer electrolyte	^[^ ^186^ ^]^
5.	Gemini‐type basic morpholine ionic liquid ([Nbmd][OH])	Chitosan	70 °C	Anion exchange membrane in fuel cell	^[^ ^187^ ^]^
6.	1‐Ethyl‐3‐methylimidazolium trifluoromethanesulfonate [EMIM][Tfo]	Hydroxylethyl cellulose (HEC)	–	Supercapacitors	^[^ ^188^ ^]^
7.	[BMIM][BF_4_]	Sulfonated poly(ether ketone) [SPEEK]	–	Fuel cell	^[^ ^189^ ^]^
8.	*N*‐butyl‐*N*‐methylpyrrolidinium bis(trifluoromethanesulfonyle) imide (C_4_mpyrNTf_2_)	LiNi_0_._5_Mn_1_._5_O_4_ (LNMO)	–	Cells (as electrolyte)	^[^ ^190^ ^]^
9.	[EMIM)[TfO]	–	–	Dual‐graphite battery (DGB) electrolyte	^[^ ^148^ ^]^
10.	[EMIM)[TfO]	Poly(vinylidene fluoride‐cohexafluoropropylene) (PVdF‐HFP)	Up to 340 °C	Sodium‐ion batteries	^[^ ^152^ ^]^
11.	[*N,N*‐bis(4‐ (methoxycarbonyl)benzyl)‐*N*‐methyl‐d‐glucaminium bromide (MBMG‐Br)	CNTs‐supported iron phosphide (FeP_(MBMG)_/CNTs)	–	Electrocatalyst for HER	^[^ ^174^ ^]^
12.	7‐Methyl‐1,5,7‐triazabicyclo[4.4.0]dec‐5‐ene (MTBD)	Lithium salt of bis(trifluoromethylsulfonyl)imide (bmsi)	–	ORR	^[^ ^176^ ^]^
13.	1‐Ethyl‐3‐methylimidazolium bis(trifluoromethylsulfonyl)amide or EMIM‐NTf_2_	Poly(IL)–customized rGO (PIL:rGO)	–	Electrode for supercapacitors	^[^ ^191^ ^]^
14.	1‐Methyl‐1‐propylpyrrolidinium bis(trifluoromethyl sulfonyl)imide ([PMpyr][NTf_2_])	PVDF‐hexafluoropropylene) (PVDF‐HFP)	–	Electrolyte membrane for supercapacitor	^[^ ^192^ ^]^
15.	1‐Methyl‐3‐(3‐trimethoxysilylpropyl)imidazolium chloride)	Titanate nanotubes (TNTs)	–	Anion exchange membrane in fuel cells	^[^ ^193^ ^]^
16.	1‐(4‐Sulphobutyl)‐3‐vinylimidazolium trifluoromethanesulphonate [HSO_3_‐BVIm][TfO]	Methyl methacrylate (MMA) and perfluoro‐3,6‐dioxa‐4‐methyl‐7‐octene sulfonyl fluoride (hPFSVE),	–	Proton exchange membrane fuel cell	^[^ ^194^ ^]^
17.	Bis(2‐ethyl hexyl) ammonium hydrogen phosphate [EHNH_2_][H_2_PO_4_], and imidazolium hexanoate [Im][Hex]	Poly(vinylidene fluoride) [PVDF]	–	Fuel cell	^[^ ^195^ ^]^
18.	Diethylmethylammonium trifluoromethanesulfonate [Dema][TfO]	PTFE	–	OER/HER	^[^ ^196^ ^]^
19.	2‐Butylaminoimidazolinium bis(trifluoromethylsulfonyl)imide (BAIM‐TFSI)	Polyimide (PI) Matrimid	–	Fuel cell	^[^ ^197^ ^]^
20.	Diethylmethyl ammonium trifluoromethanesulfonate ([dema][TfO])	Polybenzimidazole	Up to 250 °C	Polymer electrolyte for fuel cells	^[^ ^37^ ^]^

The literature reported in this section upon ORR discussed the IL‐based material's catalytic performance and applications. Also, it examined the additional documented IL‐based polymer materials, and they are multifunctional in applications. Table 3 summarizes the ILs‐based polymer composite in energy storage devices.

#### 4.2.7 Dye‐Sensitized Solar Cells

The dye‐sensitized solar cells (DSSCs) provide a technically and reasonably realistic alternative for presently used p–n junction photovoltaic appliances. DSSCs are made up of a porous dye‐sensitized nanometer TiO_2_ layer with pores of nanometer size. The electrolyte is composed of iodide/tri‐iodide (I^−^I_3_^−^) reduction–oxidation couples, and due to its lower production costs and potential, it represents an alternative to the conventional photovoltaic devices that have attracted significant attention worldwide. The DSSC are made up of liquid electrolyte having organic solvent, i.e., acetonitrile; the latter provides a total light‐to‐electricity change potential of about 11% below the illumination of AM 1.5.^[^ ^198^ ^]^ On the other hand, liquid‐intersection cells do not have a long duration, mainly due to evaporation, the outflow of organic solvent is present at high temperatures, and the sealing of the liquid‐intersection cells is not easy. To overcome these issues, numerous efforts has been dedicated on substituting liquid‐based electrolytes with ILs,^[^ ^199^ ^]^ solid or QSS electrolytes together with polymer gel electrolytes (PGEs),^[^ ^200^ ^]^ p‐type semiconductors,^[^ ^201^ ^]^ and organic conductive substances.^[^ ^202^ ^]^ Furthermore, ionic gels, as well as ionic polymeric electrolytes have been synthesized with the introduction of IL electrolytes within matrices; these composites have been investigated to avoid the outflow and provide a reliable vaporization process for the electrolytes.^[^ ^203^ ^]^ A new IL PGE having BMIM iodide, PVP, KI, and I_2_ has been synthesized and characterized. By controlling the BMIM iodide, I_2_, and KI concentration with 0.9, 0.12, and 0.5 m, respectively, the IL based polymer PGE exhibits the highest IC (at 30 °C) of 2.3 mS cm^−1^. An IL PGE with QSS in DSSC was prepared, and a total light‐to‐electricity exchange efficiency of 5.41% was obtained under the illumination of AM 1.5 (100 mW cm^−2^). The DSSC employing the IL PGE shows a much more durable photovoltaic activity compared to a DSSC consisting of liquid electrolytes (10.1080/15567030902804764). A new composite polymeric gel consisting of RTIL, (BMIMPF_6_), and heteropoly acids (phosphor tungstic acid, PWA) in a matrix of poly(2‐hydroxyethyl methacrylate) was effectively synthesized and used as an electrolyte in QSS in DSSCs. This composite polymer‐based electrolyte presented precise profit as compared to the IL and heteropoly acids that efficiently robust the IC of the composite based polymer electrolyte. This composite‐based polymeric electrolyte represents an excellent option for formerly known hole transporting substances to prepare durable QSSs, or solid‐state DSSCs. DSSCs are striking solar cell alternative due to their comparatively less preparation cost and ordinary method techniques. A recent study has shown that photoanodes were manufactured using TiO_2_ NPs (TNPs), TiO_2_ nanowires (TNWs), and its diverse composites (TNPWs). TNPs and TNWs were synthesized by sol–gel/hydrothermal process and blended at different quantities along with the ratio of their weight, i.e., (100 wt% TNPs with no TNWs), (50 wt% TNPs with 50 wt% TNWs), (90 wt% TNPs with 10 wt% TNWs), (10 wt% TNPs with 90 wt% TNWs); (whole weight 5 g). By using cobalt sulphide decorated electrode, DSSCs were developed having an IL PMII (1‐propyl‐3‐methylimmidazolium iodide) as electrolyte which was based on Indigo dye‐sensitized photoanodes. Photovoltaic investigations show that DSSC‐4 prepared by using composite photoanode (10 wt% TNWs+90 wt% TNPs) had the greatest phototransformation capacity of 2.27% in comparison to DSSC‐2 (produced from purest TNPs photoanode; *η* = 1.00%) as well as DSSC‐3 (purest TNWs photoanode; *η* = 0.77%). EIS shows that composite TNPWs photoanodes are advantageous for improving the transport of electrons and having higher charge accumulation capability and light harvesting, which result into an improvement of the photoconversion efficiency of DSSCs.^[^ ^204^ ^]^

In the entirely known DSSCs that are in solid‐state, the electrolyte that is free from iodine is poly(1‐alkyl‐3‐(acryloyloxy)hexylimidazolium iodide), which is a polymeric IL are used having an on the whole change competence of 5.29% with 1.5 AM simulated solar light (100 mW cm^−2^) enlightenment.^[^ ^205^ ^]^ The acidic polymeric IL, i.e., P[((3‐(4‐vinylpyridine) propane sulfonic acid) iodide)‐*co*‐(acrylonitrile)] and it is shortly known as P‐HI, had been used in IL electrolyte in DSSCs. The new acidic IL polymer/IL composite based polymeric electrolytes do not have iodine, and the activity of these electrolytes has been assessed by inspecting the materials of P‐HI and the potency of iodine on the activity of DSSCs. At 100 mW cm^–2^, the overall energy conversion potential of the cell depends on the electrolyte having 20 wt% P‐HI is 6.95% under AM 1.5 irradiation.^[^ ^206^ ^]^ By using 1,6‐hexanediol diacrylate in 1‐hexyl‐3‐methyl imidazolium iodide as precursor materials and by means of in situ photopolymerizations a novel type of QSS electrolytes for DSSCs had been synthesized. The conductivities of the electrolytes obtain the most favorable ratio of polymer/IL. An oligomer, namely, polyethylene glycol dimethyl ether (PEGDME) incorporated to robust the polymer and IL. By adding an adequate quantity of PEGDME to the electrolyte, the DSSCs use an electrolyte that is present in a solid state and can show 6.5% of light energy‐to‐electrical energy change potential in 41 mW cm^−2^. The in situ photopolymerized electrolyte display better constancy followed by 1000 h heat test continuing durability test.^[^ ^207^ ^]^ A composite gel‐based electrolyte, namely, poly(IL)/IL/GO synthesized, which is used in DSSCs and consists of poly(BVIM)(TFSI), 1‐propyl‐3‐methylimidazolium iodide, and GO, with no volatile organic solvent. The conductance of the composite‐based electrolytes is appreciably improved addition of a suitable quantity of GO. The DSSCs made up of composite electrolytes with GO show superior power alteration potential and improved durability than those which do not contain GO. The disadvantages of volatile liquid‐based electrolyte have been overcome by the better durability of the DSSCs, and also give various realistic techniques for the manufacturing of convenient applications for future technology.^[^ ^208^ ^]^ By the addition of GO with 1‐(3‐aminopropyl)‐3‐methylimi‐dazolium bromide, IL‐GO is synthesized, which is followed by the exchange of anion TFSI^−^. Eco‐friendly IL‐based composite electrolyte for DSSCs devoid of volatile organic liquid is synthesized by using IL‐GO and 1‐propyl‐3‐methylimidazolium iodide (PMII). Introduction of adequate quantity of IL‐GO is appreciably improved the exchange potential of DSSCs. These outcomes show that the DSSCs made up of IL‐GO/IL composite‐based electrolytes could again defeat the disadvantages of volatile liquid electrolytes and recommend practicable techniques to prepare DSSCs for future realistic applications.^[^ ^209^ ^]^ Many imidazolium‐containing IL copolymers have been fabricated by using free radical copolymerization's of monomer (MEBIm‐I), acrylonitrile, and poly(ethylene glycol) methyl ether methacrylate (POEM), which is an imidazolium‐containing IL at diverse molar ratio. A systematic study had been done to learn the impact of the chemical constitution on the IC, catalytic performance, and ionic diffusion coefficients (IDCs) of the IL‐based copolymers. The POEM‐consisting IL‐based copolymer conductivities, as well as IDCs, increase with the existence of the ethylene oxide. The introduction of appropriate amounts of acrylonitrile part increases the catalytic performance of the IL‐based copolymers. The EIS and photovoltaic characteristics of DSSCs arranged from these electrolytes that were based on IL copolymer had been assessed. A largest energy exchange potential of 7.57%, with a 16 mA cm^−2^ of short‐circuit current density, a fill factor of 57.0%, and 0.84 mV of an open‐circuit voltage had been obtained for IL copolymers‐based DSSCs (structural design: fluorine‐mixed with tin oxide (FTO) glass/TiO_2_/N719 dye/IL polymer‐based electrolyte/Pt/FTO glass) under 1.5 AM irradiation with a power of 100 mW cm^−2^.^[^ ^210^ ^]^

This section has provided an overview of the IL‐based materials DSSCs and the different reported material performances. The section also provides researchers with information to develop novel IL‐based materials for energy‐based devices.

### 4.3 ILs‐Based Polymer Nanocomposite for Biomedicine Applications

The introduction of ILs within the polymer‐based matrix had significant and valuable advantages in biomedical applications, specifically by the involvement of ILs, which provides ions, i.e., cations and anions, to provoke the cell. As a result, this allows, in some cases, under the influence of an electrical stimulus, the separation of various ions according to their potential or working power, adhesion of cells outside the body, and growth of cells.^[^ ^211^ ^]^ The utilization of polymer‐based composites in biomedicine is because of characteristics like well‐suited mechanical strength, biodegradable nature, biocompatibility, compactness, and bio‐reabsorb ability. Biopolymer‐based substances can elegantly copy the outer structural properties of the living system due to their biocompatibility.^[^ ^212^ ^]^ The field of ionic liquid‐based polymer composite includes applications in various fields like medical equipment's, tissue engineering, orthodontist applications, oral tissues, immobilization of protein, delivery of drugs to the target organ, rejuvenation medicine, bones and ligament uses, blood vessels, antimicrobic substances as well as surgical implants.^[^ ^213^ ^]^ Some of the applications are discussed in the subsequent sections.

#### 4.3.1 Bones

**Figure 25:**
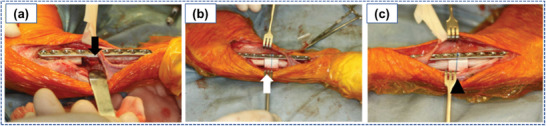
Dog's tibial bone surgery image. a) The middle portion of the tibia bone of dog's leg was detached by an oscillating bone saw (white arrow). b) The biocomposite HA/PDLLA was put at the removed bone portion (black arrow). c) The 𝛽‐TCP biocomposite was put into the empty space. Reproduced with permission.^[^ ^217^ ^]^ Copyright 2015, Hindawi Publishing Corporation.

Bones make the outer structure of the living body by making it skeletal, which provides support and safety to delicate organs. Bones consist of nanocrystals of hydroxyapatite (HA), collagen fibers, cells of bone, blood vessels, and mucopolysaccharides. HA is progressively employed for grafting and as fillers of bone to repair fractured bone and can be obtained from the animal's waste bones.^[^ ^214^ ^]^ While using these materials in biomedical, viable and environmental factors are also considered. Therefore, an environmentally viable scheme for the preparation of HA has been designed and used to manufacture HA powders (HAps).^[^ ^215^ ^]^ At present, HAps are synthesized from flora and fauna by means of total present industrialized technology. Innate and man‐made biodegradable polymer‐based composites are broadly used as scaffolds in repairing of bone because of their outstanding biological as well as mechanical characteristics.^[^ ^216^ ^]^ The research of Akagi et al.^[^ ^217^ ^]^ compared the effectiveness of HA/poly‐d/l‐lactic acid (HA/PDLLA) and *β*‐tricalcium phosphate (*β*‐TCP) scaffolds employed for implantation as well as repairing of bones. From this result, they concluded that HA/PDLLA composite based scaffolds, while using in the implantation of bones, give excellent bone repairing results due to their new bone formation mechanism, biodegradable nature, less probability of left scaffold at the end of the process with the composite materials as opposite to *β*‐TCP‐based scaffold, and its better direct migration of cells from their sources of origin reveal by immune histochemistry staining. This outcome was collected when surgery was done on both feet of the dog, i.e., right and left, in which some part of dog's tibia bone was substituted by HA/PDLLA and *β*‐TCP scaffolds, as shown in **Figure** **25**.

#### 4.3.2 Skin

Skin is the essential part of the body; it protects the inner body part from harsh outer conditions, regulates body temperature by sweating, or acts as the first immune organ of the body by preventing the entrance of any pathogen in the body. During these works, skin faces many severe conditions like skin burning, fungal infections, etc., which leads to skin damage. So, skin regeneration is a severe matter that is overcome by using ILs based polymer composites that are biocompatible and biodegradable. Many researchers have worked on this process for a long time and have given valuable results. Polymer‐based hydrogels are drug molecule carriers like anticancer, antibiotic, and antifungal drugs.^[^ ^218^ ^]^ Polymer‐based substances are also used for dressing of injury, which gives an outer covering to injured places, which helps to faster the healing process and help in tissue engineering to regenerate missing or smashed tissues by easy growth of new cells.^[^ ^219^ ^]^

On the other hand, some stretchable ILs‐based sensors have been manufactured and used to identify the wrist pulses and are compatible with money‐making rubber bands hence woven into ornaments to recognize the signal of hands.^[^ ^220^ ^]^ These biocomposite based sensors exhibit excellent conduction in the field of sensitivity. They can also identify the cervical gestures by using various wireless gadgets.^[^ ^221^ ^]^ Ionoskins are skin‐based wearable electronic strain sensors based on ionic conduction; they are in essence used for this biomedical purpose with some polymer‐based gelators, namely, [poly‐1‐(methyl methacrylate‐ran‐butyl acrylate), the IL [EMIM][TFSI] and [PMMA‐*r*‐PBA]. These gelators or ionic conductors are transparent and ultrastretchable in nature.^[^ ^222^ ^]^

#### 4.3.3 Drug Delivery

Drugs are the chemicals that we take to cure a particular disease.^[^ ^223^ ^]^ Drugs are of many types like analgesics, antipyretics, antibiotics, etc. We take drugs orally or by injection, but they always go at their target organ and provide relief. However, the drugs based on peptides and proteins are degraded by the stomach's acidic medium, and various biocatalysts present inside the intestine. To overcome this problem, we require some drug delivery agents which properly deliver drugs. An IL monomer, namely, 3‐methyl‐1‐methylimidazole and 2‐chloroethyl methacrylate, these IL monomers interject inside the montmorillonite membranes and then copolymerized through methacrylic acid. Naproxen, as a representation of medicine was trapped in nanocarriers that were pH‐sensitive and carried a positive charge, the in vitro discharge study was recognized independently in the two fluids that are free from enzymes enzyme, i.e., simulated gastric fluid (SGF with pH 1) and simulated intestinal fluids (SIF with pH 7.4). SIF has a greater drug release percentage. As a result, the synthesized nanocomposite could be regarded as an appropriate delivery agent for colon precise anionic drug delivery.^[^ ^224^ ^]^

A pH‐sensitive cationic silica NP was prepared using imidazole‐based IL for the controlled release of methotrexate. This graft copolymerization method is used, and vinyl functionalized silica NPs, as well as methacrylic acid, are used as a monomer. The above‐explained nanosystem is personalized for an anticancer agent, namely, methotrexate (MTX), for its targeted delivery. The anionic part of MTX carboxylic acid and the transporters cationic rings shows the electrostatic force of attraction. As a result, the nanocomposite‐MTX intricate was produced at a physiological pH (7.4). At the same time, the discharge of which can be obtained by the partition of the complex of nanocomposite‐MTX by the addition of a proton to carboxyl groups of the MTX part that are sensitive to changes in outer pH at weakly acidic situations. The obtained outcomes may unlock the potential for pH‐sensitive specific delivery of MTX to the carcinogenic cells.^[^ ^225^ ^]^ Drug delivery systems also depend on ILs, active pharmacological ingredients (API), and polymers. For example, a drug transport system of these kinds was prepared that depends on mefenamic acid (MEF), which is sparingly soluble in water, ammonium‐based ILs, and grafted poly(l‐lactide) (MEF–IL–LA). By using the emulsion solvent evaporation method, the NPs of MEF–IL–LA were prepared. The shapes of NPs are sphere‐shaped and 279 ± 3.6 nm to 453 ± 4.9 nm of controlled size having the greatest encapsulation potential up to 92.0 ± 2.7%.

**Figure 26:**
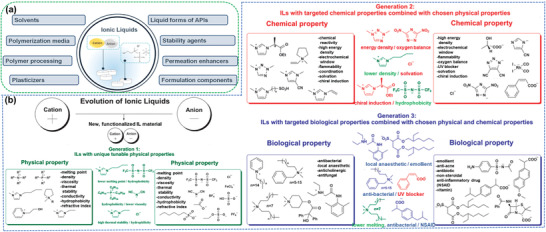
a) Implement ILs in drug transport technique's structure and consequence (APIs: active pharmaceuticals ingredients). b) Development of the scientific direction on ILs from individual material via outstanding chemical and biological property groups Reproduced with permission.^[^ ^226^ ^]^ Copyright 2021, MDPI.

Limited and constant liberation was attained up to 120 and 168 h, based on substituent of the alkyl chain and the hydroxyl ethyl groups in the compound, respectively.^[^ ^226^ ^]^ ILs had been employed with functionalized biopolymers in the transport of drug to create delivery methods and medicine preparations (**Figure** **26**a). Figure 26b illustrates the three stages of the evolution of ILs. The first class of ILs was developed by Paul Walden in 1914, and other ILs are documented within the 1970s–1980s.

A green preparation of CNT‐reinforced molecularly imprinted polymer composites has been developed by researchers for the drug delivery of Fenbufen (FB). The straightforward synthesis of single‐walled CNTs (SWCNTs)‐doped molecularly imprinted polymer (MIP)‐based nanocomposite dual green porogen arrangement, RTILs, and DESs was established for drug transport mechanism. With FB as a prototype compound, 4‐vinyl pyridine (4‐VP) was utilized as a working monomer; ethylene glycol dimethacrylate acted as a cross‐linked monomer, 1‐butyl‐3‐methylimidazolium tetrafluoroborate and choline chloride/ethylene glycol as a dual eco‐friendly solvent in the presence of SWCNTs.^[^ ^227^ ^]^

#### 4.3.4 Tissue Engineering

Due to their multifunctional behavior, ILs have been used in tissue engineering (TE) techniques, i.e., scaffolds and artificial muscles. In this way, various types of prospective and composite materials based on ILs have been known from time to time. In exacting, ionic electroactive (EA) layers that made up of ILs and PVDF was prepared by using two dissimilar kinds of ILs, namely, [BMIM][Cl] and [Ch][DHP], presented that its incorporation in to the matrix of polymer initiate the crystallization process of PVDF's polar *β*‐phase and hence enhance the electrical conductivity of the layer.^[^ ^228^ ^]^ In the two proposals, nearly 20 wt% of IL increased for the generation of cells of muscle, showing their pertinency for muscle's rejuvenation. Similarly, EA electrospun fiber composites have been developed with 1‐ethyl‐3‐methylimidazolium bis(trifluoro methane sulfonyl)amide ([EMIM][TFSI]) and PVDF.^[^ ^229^ ^]^ The phase crystallization of the PVDF fibers was also initiated by this and hence enhances the ability to live of cell with not affecting the outer structure of myocytes. ILs also have the utility for the dissolution of various proteins, like fibroin of silk, collagen (bones, muscles, tendons, skin, etc.) and keratin (nails, hairs, skin, etc.) to get scaffolds for TE utility. [BMIM][Cl] was cast‐off with fibroin, a silk protein, to obtain sequentially arranged thin layers,^[^ ^230^ ^]^ and it was exposed to favor the expected propagation and distinction of prime keratin cells. Despite this, a similar IL was utilized to dissolve keratin protein, indicating that the removal of murine embryo fibroblast was quicker while prepared in this mixture scaffolds.^[^ ^231^ ^]^ Various type of IL was used for the dissolution of collagen protein. [EMIM][Ac] was one of them, the biocompatibility of whose established with the proliferation and adhesion method of a fibroblast.^[^ ^232^ ^]^

On the other hand, for the dissolution of collagen protein, triethanolamine acetate ([TEA][A]) was also utilized. Following the dissolution, the mixture was added to the solution of Na alginate and hydroxyapatite to yield pellets to rebuild osteological disorder, raising a scheme of active bone fillers to regenerate bones.^[^ ^233^ ^]^ Similarly, to dissolve sucrose acetate isobutyrate as well as chitin an IL 1‐butyl‐ imidazolium acetate ([BMIM][OAc]) was used, which is utilize for the production of spongy form for TE and creating scaffolds having diverse pore value and with no toxic consequences on cells.^[^ ^234^ ^]^ For advancements in TE in bones, chitin as well as chitosan was also dissolved in IL which is done with mixing of it with hydroxyapatite.^[^ ^211^ ^]^ [EMIM][Ac] was utilized for the dissolution of chitin in neural TE techniques and made an even dispersal of CNTs,^[^ ^235^ ^]^ indicating that for the inspiration and restoration of nerve cells, the fabricated composite scaffolds can be used as replaceable electrodes. ILs were also used for the dissolution of a number of polymers, such as cellulose in [BMIM][Cl], along with proteins to get highly porous scaffolds.^[^ ^236^ ^]^ The formed scaffolds proved the nonexistence of cell toxicity, which is a capable opportunity for TE techniques. For the manufacturing of cellulose‐based scaffolds, an IL 1‐*n*‐allyl‐3‐methylimidazolium chloride ([AMIM][Cl]) IL acts as a solvent by using the sodium chloride (NaCl) leaching process with BSA.^[^ ^237^ ^]^ The ability to perform appropriate host responses and biological performance of the scaffolds were shown and as the capability of mesenchymal stem cells to join the formed scaffolds surface. Hydrogel concepts are also used with ILs for TE techniques, to repairing heart tissues,^[^ ^238^ ^]^ for rejuvenation of derma^[^ ^239^ ^]^ and transplant functions.^[^ ^240^ ^]^

To show the development and in vitro 3D enclosing of chief cardiac muscle cells and hearts fibroblasts, a hydrogel, namely, GelMA/Bio‐IL was established. The ability of the cell to survive and its metabolic activity were examined by the dispersion of F‐actin/DAPI staining. It was proved that hydrogels, namely, GelMA/Bio‐IL displayed a remarkably advanced ability to survive compared to the typical unadulterated hydrogels, i.e., GelMA. Formerly explained, hydrogel was utilized to produce conductive and adhesive heart patches.^[^ ^238^ ^]^ The prepared patch sticks very well to the murine myocardium by the electrostatic force of attraction between IL and tissue by excluding the requirement of suture for dermal TE utilizations was also obtained by the use of [BMIM][Ac]]. The in vitro study showed that the synthesized hydrogels could assist the adhesion and development of skin fibroblasts. The functional as well as nonliberating konjac glucomannan (KGM), which is an antibacterial hydrogel, and 1‐vinyl‐3‐(3‐aminopropyl)‐imidazolium tetrafluoroborate ([VAPim][BF_4_]) have also been prepared and utilized for the advancement of wound healing for diabetics.^[^ ^241^ ^]^

On the other hand, hydrogel‐based collagen and [EMIM][Ac] were manufactured for TE as well as for cancer treatment.^[^ ^242^ ^]^ The outcomes displayed that the HepG2 and MKN45 cells (carcinogenic cells) scattering reduced much quickly in these hydrogels compared to the blank control, i.e., no further hydrogel cancer cells.

**Figure 27:**
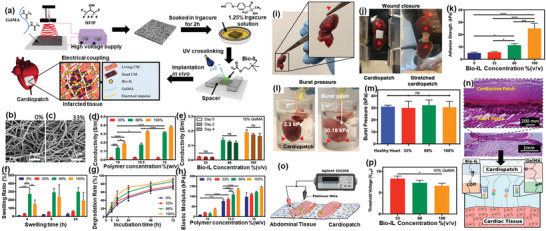
Amalgamation and material characteristics of electrospun GelMA/Bio‐IL cardio reinforcements. a) Graphic of GelMA fibrous rugs’ electrospinning was observed with absorbing within Irgacure media and Bio‐IL accumulation preceding to photo‐cross‐linking through UV rays around 5 min to construct patches. Illustrative SEM pictures of patches created by employing 10% GelMA with b) 0%, and c) 33% (v/v) Bio‐IL. d) The electrical activity of cardio patches manufactured through contrasting GelMA and Bio‐IL absorptions shows that patches’ electrical performance improved concomitantly while simulated with elevated concentrations of Bio‐IL. e) The electroconductive effects of cardiac patches after gestation within DPBS at 37 °C for 2 and 4 days showed that electrical performance did not reduce. f) Inflammation proportion, g) dilapidation rate in collagenase class II media over the period, and h) flexible modulus of manufactured cardio covers GelMA was utilized. Ex vivo Glue effects and electrical activity of GelMA/Bio‐IL cardio covers. i) An illustrative manifestation of a GelMA/Bio‐IL cardio reinforcement image cross‐linked upon an explanted rat heart shows the cardio cover's better sticking (red projectiles) to heart tissues. j) Ideal injury closing trial utilizing explanted heart of rat as the natural support to experiment with the adhesion power of GelMA/Bio‐IL cardio patches. k) Quantifying the bonding force indicated through cardio covers manufactured via 10% (w/v) GelMA and variable Bio‐IL amount upon explanted rat hearts. l) Illustration photos of GelMA/Bio‐IL cardio patch manufactured through 10% (w/v) GelMA and 66% (v/v) Bio‐IL photograph cross‐linked upon the deficiency spot of an explanted rat heart to estimate the rupture pressure. m) Quantification of the combustion pressure of GelMA/Bio‐IL cardio coverings constructed through variable absorptions of Bio‐IL and image cross‐linked upon the imperfection position of rat heart revealed no important distinction than the combustion pressure of a fit mice heart. n) H&E staining of cardio patch‐tissue edges. o) Schematic of ex vivo intestinal tissue positioned adjacently upon GelMA/Bio‐IL cardio reinforcements manufactured through 10% (w/v) and unstable concentrations of Bio‐IL to define the point voltage required to enable each section of intestinal tissue. p) Quantification of the verge potential of GelMA/Bio‐IL cardio patches extremely reduced toward patches manufactured by 100% (v/v). Reproduced with permission.^[^ ^241^ ^]^ Copyright 2019, Elsevier Ltd.

Electrospun patches were set within 1.25% (w/v) Irgacure 2959 into ethyl alcohol around 2 h, heeded through direct acquisition of different absorptions of Bio‐IL and cross‐linking by the direction toward UV rays for 5 min (**Figure** **27**a). To describe the fiber topology of GelMA/Bio‐IL cardio patches were prepared through variable absorptions of Bio‐based IL and through scanning electron microscopy (SEM) (Figure 27b). The outcomes demonstrated that the electronic performance of the platforms would be adjusted by changing the engagements of each GelMA and Bio‐IL (Figure 27c). All findings indicated that the electronic performance of GelMA/Bio‐IL cardio reinforcements showed no statistically essential distinctions even after 4 days of gestation toward all circumstances tried (Figure 27d). The outcomes demonstrated that the conductive effects of the platforms were not influenced via the breakdown (Figure 27e). Additionally, the results indicated that staging's incorporated through 10% (w/v) GelMA and variable Bio‐based IL absorptions surged quickly after 4 h of gestation, without substantial water uptake growth after 8 and 24 h toward all Bio‐based IL concentrations (Figure 27f). The dilapidation rate improved concurrently while the Bio‐IL absorptions were raised for cardio reinforcements possessing 10% (w/v) GelMA (Figure 27g). Therefore, the mechanical effects of staging's incorporated are estimated utilizing a variable amount of GelMA and Bio‐based‐IL (Figure 27h). Preferably, wound closure experimentations were accepted to consider the sticky power of the scaffolds to porcine derma and murine left ventricular heart muscles (Figure 27i,j). It also assessed the capability of cardio patches of GelMA/Bio‐IL to pack tissue imperfections below involved pressure utilizing collagen films established upon a typical burst pressure trial and ex vivo explanted rat hearts (Figure 27k–m). The intense adhesion of GelMA/Bio‐IL cardio reinforcements to the heart muscles was also demonstrated through the histological development of the patch and tissue (Figure 27n). For this, the muscles, namely, rectus abdominus of Wistar rats, were explanted autopsy, slashed within square parts, and put 3 mm separated from each other on the lid of the staging's (Figure 27o). As predicted, the findings revealed that frameworks with higher Bio‐IL concentrations showed comparatively lower point voltages than GelMA patches in the absence of Bio‐IL (Figure 27p).^[^ ^241^ ^]^

#### 4.3.5 Cancer Therapy

To fight carcinogenic cells, the development of ILs provides an indispensable role. For example, novel advances in sensors used to recognize biomolecules at very low concentrations have come to light. In the manufacturing of pharmaceuticals remedy, it is utilized in their preparation. It works like a solvent enabling its sanitization or permitting its withdrawal from naturally obtained materials and allowing their steadiness. Transport of medicines can act as a transporter, accessory, or unswervingly as an active API.^[^ ^243^ ^]^ As we know, the exclusive characteristics of ILs behave as an eco‐friendly solvent, like lower vaporization rate, the initial and many profuse uses of ILs are for solubility of cancer‐treating drugs. The use of ILs resolves numerous difficulties related to anticancer drugs. Much of the anticancer drug's nature is crystalline solid, showing some related problems like polymorphism and less bioavailability.^[^ ^244^ ^]^ The decent solvation abilities and IL physical and chemical alteration have been utilized, for illustration, to open the novel, more complicated pathways to the transport of the medicines like topical transport, which is known as the best path for therapy of exterior tumors like melanoma.^[^ ^245^ ^]^ The more significant figure of probable salts that are ionic makes it to be very clear that a wide variety of well‐admired characteristics can prepare ILs, therefore, making it capable of finding better runners with anticarcinogenic characteristics, i.e., act as APIs. This is much significant that the earlier explained properties of ILs as eco‐friendly solvents are no longer apparent, as their biological relations have been proved in many types of research.

Though these have some shortcomings in biotechnology and the environment, these properties are extremely significant in treatments of cancer where cell toxicity is initiated in carcinogenic cells. In that manner, a wide‐ranging organization of investigational outcomes had previously been attained by meant for the preferable ILs concerning its cell poisoning for some cancer‐causing cells. By giving an illustration, the crucial part of water‐hating and lipids loving the cations has been found.^[^ ^246^ ^]^ A surge in the acyclic side part of imidazolium, phosphonium, ammonium, and pyridinium based cations has been noticed as an enhancement in cell poisonousness. By contrast, the existence of oxygen on the substituted chain reduces it.^[^ ^247^ ^]^

This had been accredited to the inter attraction and addition of the cation inside the membrane of cell.^[^ ^248^ ^]^ Symptoms like the failure of mitochondria, stress based on oxidation, and programmed cell death have also been detected in carcinogenic cells.^[^ ^249^ ^]^ In the field of remedial therapy of cancer, the purpose is to enhance the testified poisonousness upon the cancerous cells by minimizing the side effect on a normal group of cells. This dissimilarity in cell toxicity cooperating by means of enhanced permeation retention consequences is the foundation of an effective treatment. Various inspiring outcomes have revealed a high need for cell poisonousness with the cell nature.^[^ ^250^ ^]^ In this manner for the cells of healthy human embryonic kidney and cancer of brain (T98G), imidazolium, and ammonium based four ILs had been detected, namely, 1‐methylimidazolium chloride ([MIM][Cl]), triethylammonium hydrogen sulfate ([TEA][SO_4_H]), [BMIM][Cl], and triethylammonium hydrogen phosphate ([TEA][PO_4_H]).^[^ ^251^ ^]^ Noticeably, when the ILs used in a concentration of more than 0.01 mg mL^−1^ show less toxic behavior over normal cells but major toxicity toward cancer causing cells, [BMIM][Cl] displayed the maximum anticancer performance. With the utilization of additional traditional ILs like APIs and appreciation of their resourcefulness, it may also be conceivable to change various medicines into an IL (API‐IL) directly. Egorava et al.^[^ ^252^ ^]^ defined three routes to change any drug into an IL. The first method involves using a remedy that is ionic in nature with the collaboration of such ions which carry an opposite charge, such as cationic IL, namely, imidazolium, with a negatively charged drug. The second method comprises the dative bond of the medicine with the ILs cationic part, sometimes more rarely, furthermore with the anion. The third method explains the combination of the first and second methods by using two concurrent drugs. This research described seven ILs comprising salicylic acid (SA). In addition, by an enhancement of SA solubility, the cell toxicity also increases on colorectal adenocarcinoma human cell line (CaCo‐2) associated with traditional ILs that is imidazolium‐dependent. While no significant variance in poisonousness was detected in normal cells in this instance, it unlocks the technique for the synthesis of other advanced medicines for cancer therapy.

Combining the ILs with other materials provides the best degree of function in cancer therapy. Occasionally, for the transport of medicines, various more advanced substances are synthesized by combining ILs with polymers. Cui et al.^[^ ^253^ ^]^ designed NPs by combining 1‐ethylvinylpyridinium [EVPy] based cation, i.e., poly(IL‐*co*‐N‐isopropylacrylamide) with an anionic part consist of deoxycholic acid. The shaped NPs were utilized like medicine transporters to convey the doxorubicin (DOX) that is model drug by a skillful transport by changing the temperature and pH. For the transport of 5‐fluorouracil, polyacrylate as well choline are used as nanogels.^[^ ^254^ ^]^

The IL‐based scheme displayed much constancy and a protracted medicine that release at an acidic stomach medium having pH (1.2) for an additional ten days. In a diverse case, combining polydopamine NPs with DOX and [EMIM][PF6] as model IL and anticarcinogenic drugs was known for collective hyperthermia therapy and chemotherapy. The scheme was also used to treat various disorders like tumors in rats, and, remarkably, the IL was not used to initiate poisonousness by self but as a sensitizer for radiations, i.e., microwave.^[^ ^255^ ^]^ The implementation of microwaves creates a temporary enhancement of temperature, which initiates the death of cells in tumor areas. The outcomes displayed the entire elimination of tumors in 16 days when progressive development of tumors was detected. To upgrade the characteristics of biological hydrogels, ILs are introduced.

For the development of hydrogels, [EMIM][Ac] introduced by Li and Fan^[^ ^242^ ^]^ which are prepared by the cross‐linking of microbial transglutaminase in between fishbone collagen (FBC) and genetically engineered collagen (human‐like collagen, HLC). With advanced porosity and higher mechanical strength, the [EMIM][Ac] had substantial belongings on the properties and structure of the hydrogels. Additionally, the IL occupies an important function in preventing the gel's enzyme‐based hydrolysis, which is facilitated to sustain the material purposes for a long time. The cell‐based studies were done on the gels by using well normal cancer HepG2 and MKN45 cells and normal fibroblasts 3T3‐L1 and L929 cells. The fibroblast explosion was enhanced by using the gels FBC as well as HLC. At the same time, they had a much hindering consequence on the growth of carcinogenic cells because of the ILs characteristics against the cancer and the modification of the organizational properties of the gels.

#### 4.3.6 Antimicrobial Agents

Due to their improved performance, ILs are described as the chief cause for its cell toxicity. Whereas this characteristic is problematic for eukaryotic, mammalian cells, as seen in the case of cancer treatments, it however provides excellent opportunities in microbiology.^[^ ^256^ ^]^ ILs have a unique capability of twinning with the cell walls of microbes, with which they accumulate at the layer's constituents and hence break the integrity of the cell membrane,^[^ ^257^ ^]^ which is ideal for killing microbes. This property is remarkable, since new materials/substances capable of eradicating or preventing the development of bacteria or fungi is critical to the development of bacterial and viral resistance, as the recent events related to the novel coronavirus SARS‐CoV‐2 have shown. Existing issues related to antimicrobial/viral resistance are exacerbated by the abuse of antibiotics and biomatrix or medicine contaminations through fungi or viruses. One of the remarkable properties of ILs is that they can dissolve in numerous liquids, including H_2_O, which makes ILs appropriate for biomedical implementations. Some solutions of ILs in water have been certainly known to have effective antimicrobial performance.^[^ ^258^ ^]^ While dissolved ILs are not regarded as actual ILs because they do not contain entirely of parent ions for a long time, this indicates that the required process is because of one either both ions, collectively both ions show innate performance against microbes.^[^ ^259^ ^]^

Initial in vitro research for a relationship involving the construction of the ILs and its probable characteristics against microbes had been conducted on numerous microbes like bacteria,^[^ ^260^ ^]^ redworms,^[^ ^261^ ^]^ zebrafish,^[^ ^262^ ^]^ as well as algae.^[^ ^263^ ^]^ For the biological performance of ILs, the size of cationic chains plays an important role. By accumulating on the anionic membrane of microorganisms, IL's cationic lengthy side chains form a heterogeneous spatial part. These properties made cations much sensitive constituents in the direction of cell walls of microbes, as confirmed by the remark of various physical processes of accumulation of several types of ILs with diverse cations on the surface of bacteria cells, like *Escherichia coli*.^[^ ^257^, ^264^ ^]^ In reality, IL solutions against bacteria have the same chemical arrangements as other recognized cation based chemicals that kill microbes and surface active agents, like quaternary ammonium materials, having both properties like‐charged water‐loving head part and water‐hating tails.^[^ ^265^ ^]^ These similarities show that ILs may combine to form micelles that are amphiphilic in nature inside the solution,^[^ ^266^ ^]^ whose capability is enhanced by enhancing its solubility in fats or lipids that may be operated by increasing the side chain of the alkyl group. Micelles are capable of discarding the membrane's wholeness by ionic attraction in the positively charged part of the ILs and the negatively charged part of the cell's membrane, leading to a decrease in the barrier activity of the outermost layer.^[^ ^267^ ^]^

The described process is the furthermost known process related to the use of ILs for microbes like fungi bacteria, which is chiefly credited to the cations of IL. However, cationic and anionic parts or amalgamation of these two show some activity with the cell layers. During investigations on the layer model interface of ILs in bioengineering duplications, it was found that cations or anions can interact on the bilayer of lipids, altering the organizational and changeable characteristics of the bilayer, which leads to its permeability.^[^ ^268^ ^]^ A parallel activity procedure was noticed when alkyl tributyl phosphonium chlorides were put in interaction with the fungi *Aspergillus nidulans*, harming the filaments and the wall of the cell.^[^ ^269^ ^]^ The possible poisoning of three alkyls [(1R,2S,5R)‐(−)‐methoxy methyl] dimethylammonium chlorides have also been established for various Gram‐negative bacteria (*E. coli*, *Pseudomonas aeruginosa*), Gram‐positive bacteria (*Staphylococcus epidermis*, *Staphylococcus aureus*), and wild type bacteria like *Candida albicans*, also signifying the consequence of the lengthening of alkyl substituent on their antimicrobial chain length on their antimicrobial action.^[^ ^270^ ^]^

Aside from its immediate consequence on microbes, ILs antimicrobic activity can be surged by combining some additional common biocides, showing a significant current examined center. For example, the 1‐alkyl‐3‐methyl imidazolium‐based IL had been coupled with anions having Ag and Cu to enhance the antimicrobial property in contrast to various disease‐causing bacteria and fungi.^[^ ^259^ ^]^ Differently, an antibacterial IL and a peptide that was antimicrobial in nature were coupled by “click” chemistry, producing against behaviors toward Gram‐negative, many drug‐resistance medical isolates and anti‐biofilm mechanism for an unaffected clinical *Klebsiella pneumoniae* isolate.^[^ ^271^ ^]^ To increase the antimicrobial performance of *K. pneumoniae*, *Bacillus subtilis*, and *S. aureus* and the category of some organic salts related to ciprofloxacin as well as norfloxacin are introduced as anions.^[^ ^272^ ^]^ Chi and lysozyme, which are known natural substances that work against microbes, were also coupled with various ILs to attain an extra effective antimicrobial agent. The guanylate chitosan byproducts presented a more antimicrobial nature than well‐ordered Chi.^[^ ^273^ ^]^ At the same time, when choline, deoxycholate or lauryl sarcosinate were combined with lysozyme and displayed better antimicrobial properties toward the Gram‐negative bacteria like *E. coli* and *P. aeruginosa*.^[^ ^274^ ^]^ The ILs efficiency to enhance the activity of peptides that is bioactive in nature has been lately displayed. Using “click” chemistry, synergy occurs between an antibacterial IL and an antimicrobial peptide. The obtained materials were known to keep the antimicrobial property of peptide's in contrast to Gram‐negative bacteria multidrug‐resistant clinical isolates and better stability for tyrosinase‐mediated tempering.^[^ ^271^ ^]^

Resources, as well as external surfaces made of ILs, had also been described to ignore bacteria colony formation and biofilm establishment, which is a significant aspect for missing contaminations by the contact with polluted sides. The layers made up of Imidazolium that was based on various anions, and cations have presented nature against bacteria in contrast to *S. aureus* and *E. coli*, the power of which increases by enhancing the length of the chain of cation, at a similar time being supposed to be biocompatible. On treating PVC substrates with an IL, i.e., phosphonium as an outside layer to get a super water‐loving exterior that ignored the sticking of *P. aeruginos* and *S. aureus*, also with a bactericidal consequence. Poly‐IL film that based on imidazolium and quaternary ammonium incorporated with zinc is effective on wound when tested on *S. aureus* contaminated mice by using in vivo studies of methicillin‐resistant.^[^ ^275^ ^]^

The above study shows that ILs feature various antimicrobial properties against different bacteria, algae, and fungus, but they are mostly poisonous to mammals cells. This is a disadvantage of ILs for biomedical applications. For every IL‐based antimicrobial research, the toxicity for various types of cells was also assessed, hence determining the proper concentration for their safe use. **Table** **4** shows the application of ILs‐based polymer nanocomposite in biomedical.

**Table 4:** Application of ILs‐based polymer nanocomposite in biomedical

Sr. no.	ILs	Materials	Applications	Refs.
1.	3‐Methyl‐1‐[2‐(2‐methyl‐acryloxy)ethyl]imidazolium chloride	pH‐sensitive polymer/montmorillonite (MMT)	Colon specific drug delivery system	^[^ ^224^ ^]^
2.	[EMIM][Ac]	Cellulose/Fe_3_O_4_ NPs/heparin	Magnetically responsive drug delivery	^[^ ^276^ ^]^
3.	[BMIM][Cl]	Silk fibroin	TE scaffold	^[^ ^277^ ^]^
4.	[TEA][A]	Collagen–alginate–HA	Regeneration of bones	^[^ ^233^ ^]^
5.	[EMIM][Ac]	Collagen‐based hydrogel	Treatment of cancer	^[^ ^242^ ^]^
6.	[BMIM][Ac]	Silk/chitosan‐based hydrogels	Tissue engineering of skin	^[^ ^239^ ^]^
7.	[VAPim][BF4]	KGM hydrogels	Wound healing of diabetic	^[^ ^278^ ^]^
8.	[EVPy][DA]	Poly(IL‐*co*‐nisopropylacrylamide)/doxorubicin	Breast cancer	^[^ ^253^ ^]^
9.	[EMIM][PF6]	Polydopamine	Liver cancer	^[^ ^255^ ^]^
10.	Cetpyrsal	Paclitaxel (PTX)	Ovarian, breast, and pancreatic cancer	^[^ ^279^ ^]^
11.	1‐Alkyl‐3‐methyl imidazolium chloride	–	Broad spectrum antibio layer	^[^ ^258^ ^a]^
12.	Imidazo[1,5‐a]quinoxalin‐4‐on‐1‐yl)‐1‐pyridinium bromide	–	Antimicrobial activity for yeast and gram‐positive bacteria	^[^ ^280^ ^]^
13.	[BMIM][NTf_2_] 1‐*n*‐butyl‐3‐methylimidazoilium bis(trifluoromethanesulfonyl) imide	Glycidyl methacrylate and triethylene glycol dimethacrylate	Prevent tooth decay	^[^ ^226^ ^]^
14.	Pyrrolidinium ILs	Acrylonitrile, styrene	Dermal	^[^ ^281^ ^]^
15.	Imidazolium ILs (loaded in calcium phosphate‐based nanocomposites	Calcium phosphate‐based nanocomposites	Orthopaedic	^[^ ^282^ ^]^

### 4.4 ILs‐Based Polymer Nanocomposite for Actuators Applications

**Figure 28:**
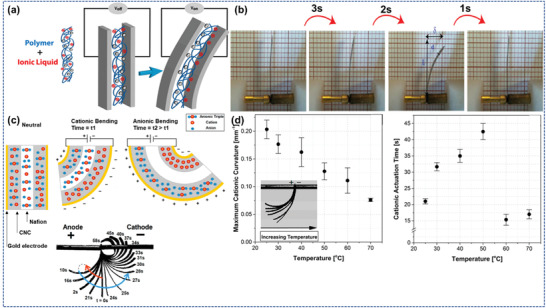
a) Graphical representation of migration of the ions and their mixing response. b) Winding gesture as a utility of period intended for the [PVDF]/[PMIM][TFSI] compound on a 100 mHz and 5 V. Reproduced with permission.^[^ ^283^ ^]^ Copyright 2019, American Chemical Society. c) Images show the bending process in the cationic and anionic way stepwise. d) Graphs show maximum cationic curvature and cationic actuation time, respectively, at diverse temperatures. Reproduced with permission.^[^ ^284^ ^]^ Copyright 2017, MDPI.

ILs‐based polymer composites have been effectively employed to form small voltage and massive, bendable electroactive actuators (EAAs). They are based on ionic polymer composite and two conductive and bendable electrodes located on both sides. On applying an electrical field, there is an ion diffusion of ions that takes place in the polymer matrix (**Figure** **28**a,b).^[^ ^283^ ^]^ As a result, a bending response is originated as an effect of the movement of charged particles, i.e., cations and anions, and their allocation nearby the electrodes. Soft actuators benefit from excellent elasticity, rapid reaction, light, less power utilization, and lower prices.

The EAAs are steadier over repetitive bending cycling and can work in a wide variety of temperatures because of the heat resistance property of ILs.^[^ ^285^ ^]^ Amongst the various EA polymers, a well‐identified polymer for its polarity, superior dielectric constant, and IC is PVDF.^[^ ^286^ ^]^ So, the actuators made up of PVDF and ILs having various anions, 1‐hexyl‐3‐methylimidazoliumchloride ([HMIM][Cl]) and 1‐hexyl‐3‐methylimidazolium bis (trifluoromethylsulphonyl)imide ([HMIM][TFSI]) had been synthesized to know the consequence of the size of anion and filled materials on the mixing behavior.^[^ ^287^ ^]^ It was assessed that polymer crystallization in the EA *β*‐phase depends on the ILs existence, not on the materials from which it is made. The electrical conductivity of a substance is based on the quantity of IL and the size of the anion. On the spot, the bending is associated with the extent of crystallinity, mechanical characteristics, and IC. The mechanical properties are almost independent of the type IL, while the conductivity is much based on the kind of anion used rather than the alkyl group's size. The effect of the width of film on the behavior of PVDF/[EMIM][TFSI] (1‐ethyl‐3‐methylimidazolium bis (trifluoro‐methyl sulphonyl) imide) was also examined, and it was found that the mix actuation of the blended materials changes appositively with the width of the sample.^[^ ^288^ ^]^

On a different manner, it is also based on the shape of the cation size.^[^ ^289^ ^]^ The effect of the matrix that was polymer‐based on the actuator activity had also been assessed with the IL, i.e., [EMIM][TFSI] inside various fluorine‐containing matrixes, communicating that the actuator execution is based on the type of polymer used.^[^ ^290^ ^]^ PVDF mixed with *N*,*N*,*N*‐trimethyl‐*N*‐(2‐hydroxyethyl) ammonium bis(trifluoromethyl sulphonyl) imide ([N_1112OH_][TFSI]) and [EMIM][ESO_4_] to know the effect of cations. The outcome exposed the mixing response is much rather based on the IL amount than its kind. A preformed polymeric membrane on perception with ILs results in electromechanical actuators. The membranes of Nf with [EMIM][TfO] as well as nanoparticles of gold act as anionic part, and its electromechanical activity is a function of its temperature of formally explained anionic part direct to actuators having less cationic curvature with an increase in temperature and the reorganization process was slow with an increase in temperature near 50 °C. While more anionic, curvature with increase in temperature was also noticed, and the speed of the reorganization was increasing at a temperature of 60 °C (Figure 28c,d).^[^ ^284^ ^]^

The functionality was lost over a temperature of 60 °C due to a variation in Nf's internal structure and the presence of some nanochannels. The movement in charges rose at 80 °C, and a sudden rise was also noticed at 90 °C. The procedure of actuation of dry‐type polymer actuators made up of SWCNT, an IL, and a polymer which acts as a base (known as nanocarbon polymer) in three layer arrangements, was also assessed.^[^ ^291^ ^]^ Good‐performance based printable polymer actuators contain the [EMIM][TFSI] IL, sulphonated polyimide that is soluble, and compounds of carbon having IC up to 1 × 10^–3^ S cm^−1^ were synthesized. The actuators have much rearrangement and a broad potential window of up to 3.5 V. The reorganization of the printable actuators was directly based on the electric charge that was assembled on the electrodes and not based on the carbon substances.^[^ ^292^ ^]^ Conductive elastomers were synthesized by combining Imidazolium‐based ILs and epoxidized natural rubber. These materials showed exciting behavior for sensors, as well as actuator‐based applications. Natural rubber is an inexhaustible substance having excellent characteristics like flexibility and outstanding mechanical property. The mechanical nature was also changed by introducing ionic liquids because of its known plasticizing outcome, retarding the strain‐initiated crystallization process in the rubber and the degree of cross‐linking changes with change in the concentration of the IL.^[^ ^293^ ^]^

The transparent actuators are prepared from the poly(dimethyl siloxane) (PDMS) and 1‐ethyl‐3‐methyl imidazolium trifluoro methane sulphonate ([EMIM][CF_3_SO_3_]) was synthesized by electrode spray casting. PDMS shows brilliant properties like flexibility, clearness, adaptable surface chemistry, less permeability for water, and low electrical conduction, and thus becomes an attractive aspirant for the synthesis of sensing and the devices, which convert energy from one form into another. The prospective of the manufactured substances as actuators stems from two methods: the electrostatic double‐layer capacitor and the Faradic capacitor mechanism.^[^ ^294^ ^]^ Some IL/polymer actuators are derived from nature and is also developed. Poly(3‐hydroxybutyrate)/1‐butyl‐3‐methylimidazoliumbis (trifluoro methyl sulphonyl) imide ([BMIM][TFSI]) known as soft actuators and are H_2_O and humidity proof having a macroscopic bending that can be reversed for giving potentials of 0.1 to 7 V and brilliant working robustness equal to 105 cycles at 2 V was begin.^[^ ^295^ ^]^ Biodegradable actuators are quick to respond to the application of low voltages.

### 4.5 ILs‐Based Polymer Nanocomposite for Environmental Applications

**Figure 29:**
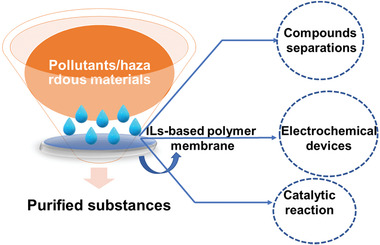
Different applications of ILs‐based polymer membrane for VOCs separations.

Over the past couple of years, the population explosion and modernization have produced considerable environmental problems. The various human actions turn out lethal pollutants like metal ions, organic chemicals, dyes, and some other inorganic materials, which are separated from water barely.^[^ ^296^ ^]^ All these substances are highly poisonous to flora and fauna; they must be removed before being released in the water bodies or the air.^[^ ^297^ ^]^ Various techniques have been developed for environmental conservation and to improve its quality; despite this, adsorption is a suitable method due to its multiple benefits like more efficiency and much selectivity, without producing sludge, easy to operate, reusable eco‐friendly, and highly economic.^[^ ^298^ ^]^ **Figure** **29** shows the different applications of ILs‐based polymer membrane for volatile organic compounds (VOCs) separations.

In addition to this, the process of adsorption may be helpful for the two mainly polluted environments in nature: water and air. The distinctive characteristics of ILs permit scheming (altering cations and anions) definite custom‐made adsorbent substances, having particular physicochemical behavior, which is similar to the nature of a contaminant. Environmental treatment can be done very effectively using sorbent materials and ionic liquid. On the other hand, IL can be used as a greener solvent in place of previously used poisonous solvents to produce materials and reduce the pollution due to the industry.^[^ ^299^ ^]^ The treatment of water and air is done by using ILs.

#### 4.5.1 Water Treatment

In recent years, water contamination emerged as one of the many unprecedented crises due to rapid urbanization and automation.^[^ ^300^ ^]^ The world health organization reported that about 6 billion people will face water starvation by 2050.^[^ ^301^ ^]^ Due to water lack and consumption, the permit for freshwater each day has different approaches to treat wastewater as the primary origin of getting to freshwater. The water remedy is being redirected to implement an efficient and economical way that enables resource retrieval.^[^ ^302^ ^]^ Therefore, the effect of distinct methods and technologies for the sanitization of polluted water is a crucial environmental issue.^[^ ^303^ ^]^ Different kinds of adsorbents, like biological/man‐made polymers and bio‐based substances, have been used to remove contaminants from polluted waters and solutions in water; the usefulness of adsorbents, including the capability to purify polluted water, is a tempting subject in water processing. Thus, assemble creative adsorbents with specific adsorption and good adsorption/desorption behavior below soft and greener circumstances.

ILs are critical in water treatment due to their higher prospect of withdrawing a broad coverage of contaminants from aqueous media.^[^ ^304^ ^]^ IL‐based antimicrobial substances are engaging nominees as adsorbents, including the double role of adsorbing the organic/inorganic impurities and discarding the microbial contaminants from aqueous media. Water pollution has been a major environmental severe problem during the past decades. Various new substances have been prepared for this problem from time to time, in which ILs are the best material to remove this problem. ILs working process is based on photocatalysis and adsorption of pollutants present in water. Many works are done on this application devoted to ILs‐based materials.^[^ ^305^ ^]^

**Figure 30:**
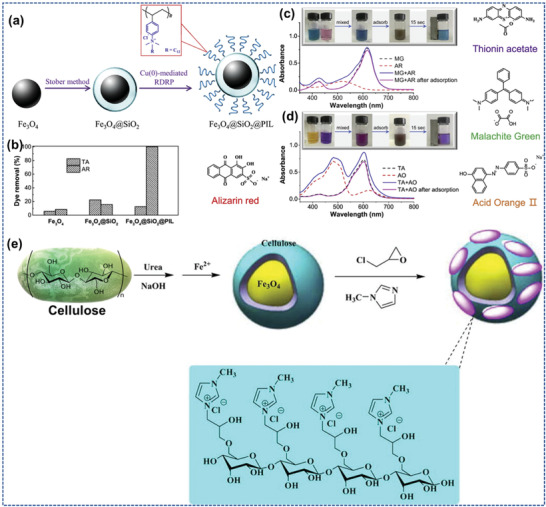
a) Synthesis pathway to Fe_3_O_4_@SiO_2_@PIL MNPs. Adsorption process to mixed and solo dyes. b) The rate of removal of AR and TA by various adsorbents materials at a primary dye amount of 50 mg L^–1^, it adsorbs Fe_3_O_4_@SiO_2_@PIL selectively. c) MG/AR with a primary amount of dye of 20 mg L^–1^. d) AO/TA on preliminary colorant absorption of 20 mg L^−1^. Reproduced with permission.^[^ ^306^ ^]^ Copyright 2019, Elsevier Ltd. e) Diagrammatic representation of the formation of Fe_3_O_4_@cellulose nanohybrid, with a new polymer‐based ionic liquid (PIL). Reproduced with permission.^[^ ^307^ ^]^ Copyright 2016, Elsevier Ltd.

Yang et al.^[^ ^306^ ^]^ synthesized sorbent material to remove water pollutants like anionic dye (**Figure** **30**a). These dyes are alizarin red (AR), malachite green (MG), thionic acetate (TA) as well as acid orange II (AO). These dyes were also removed using synthesized sorbent material, i.e., Fe_3_O_4_@SiO_2_@PIL (PIL) nanocomposite. This nanocomposite can be obtained by coating a magnetic core with silica which is further functionalized with PIL in which IL monomer unit is (dimethyl‐dodecyl‐4‐vinyl benzyl ammonium chloride). In this AR, sorption efficiency to H‐bonds, ionic bonds as well as *π*–*π* interactions. On the other hand, a repulsion between MG (cationic dye) and PIL prevents the adsorption of dye and enhances selectivity. Fe_3_O_4_@SiO_2_@PIL can adsorb AR selectively after magnetic separation of a solution of AR and MG, leaving MG in the solution (Figure 30b,c). The adsorbent would also selectively vacate AO from TA solution, as illustrated in Figure 30d, demonstrating Fe_3_O_4_@SiO_2_@PIL MNPs toward adsorption anionic pigments.

A novel ILP composite was produced by Zhu et al.^[^ ^308^ ^]^ combining a vinyl functionalized silica surface with 1‐butyl‐3‐vinyl imidazolium bromide ([BVIM][Br]). When the adsorption test was done with 2,4‐dichlorophenol (2,4‐DCP), 2,4‐dinitrophenol (2,4‐DNP), bisphenol A (BPA), etc., using various silica surfaces like vinyl silica pristine silica, it was found that the IL‐P sample adsorbed a high amount of water pollutants in less time. This shows an advanced result in the existence of IL on the surface of silica, in which ionic as well as *π*–*π* interactions occur involving the imidazolium ring (IL), which is positively charged and negatively charged aromatic ring which is related to phenolic pollutants. It is also found that ILs are reusable after treating polluted water. By combining cellulose–Fe_3_O_4_ with PIL using epichlorohydrin and 1‐methyl imidazole, a magnetic sorbent material was generated tested for an anionic dye like Congo red (CR)[307]. CR adsorption attains equilibrium after 11 min with 131 mg g^−1^ (Figure 30e).

By combining chitosan and IL (CS‐IL), natural polymer‐based materials were prepared for the adsorption of anions (PF_6_^−^, Cr_2_O_7_^−^) from the polluted water.^[^ ^309^ ^]^ IL‐hydrogels also act as sorbent material for cationic and anionic pollutants.^[^ ^310^ ^]^ Similarly, IL‐based cross‐linked polymer like polydivinyl benzene (PDVB) 1‐aminomethyl‐3‐vinylimidazoliumchloride hydrochloride IL (PDVB‐IL) was used to get rid of Cr (VI) from H_2_O.^[^ ^311^ ^]^ In which maximum work capacity is between 12 and 2 pH values.

#### 4.5.2 Air Treatment

Fast urbanization and automation for enhancing social life have obtained undesirable air pollution problems. Air contaminants, particularly CO_2_ and particulate materials (PMs), are severe environmental concerns pushing extreme harm to human fitness.^[^ ^312^ ^]^ Air pollution is detrimental to the air passageways and lungs and may harm other human body organs.^[^ ^313^ ^]^ Therefore, there have been increasing worries about decreasing or eradicating the threats originating from air impurities. Air separation processes may eliminate air impurities and enhance respiratory air grade.^[^ ^314^ ^]^ The separation composites recreate a vital part within the air infiltration method, and the invention of creative air filters has acquired significant concentration in current days. Nevertheless, traditional filtration substances are useless for dismissing microscopic particles. Therefore, air purification utilizing nanofibrous sieves and nanofibrous membranes has garnered significant favor; wildly; electrospun nanofibrous membranes have achieved importance within the percolation field owing to their distinguished effects.^[^ ^315^ ^]^

Tiny PMs, for example, bioaerosols, can have different germs, microbes, and fungi that may induce intense allergic, respiratory, and communicable disorders via communication in the air, therefore, it is essential for the vision of antimicrobial, antiviral medicines, air sieves.^[^ ^316^ ^]^ Nowadays, ILs‐based substances are among the possible prospects of air filter incorporation due to their unique gas split, photoresists, disinfectant effect, and anticorrosion, allowing a relatively unique method to evolve within the last two years. Different ILs‐based membranes have been laid for air treatment with reinforced IL membranes, polymer/IL compounds, polymer/IL/inorganic, and IL‐gelled films.^[^ ^317^ ^]^

**Figure 31:**
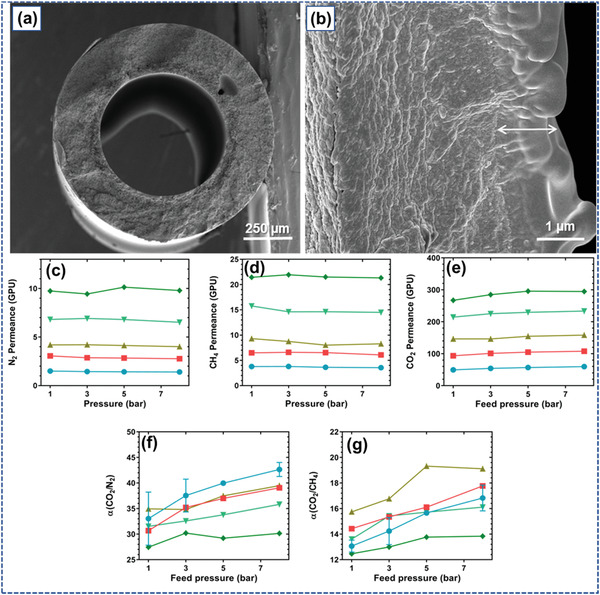
The cross‐sections show the SEM image of a) a gel membrane of hollow fiber Pebax/IL. b) Boundary among PTMSP drain coating and Pebax1657 discerning film. Consequence of feed pressure upon c) N_2_, d) CH_4_, e) CO_2_, f) CO_2_/N_2_ discrimination, and e) CO_2_/CH_4_ fussiness toward well‐ordered Pebax 1657(•), Pebax 1657/IL40% (▲), Pebax 1657/IL20% (■), Pebax 1657/IL60% (▼), and Pebax 1657/IL80% (◆). Reproduced with permission.^[^ ^322^ ^]^ Copyright 2017, Elsevier Ltd.

Among various problems of the environment, high energy utilization among global warming paid much attention to gas separation, most likely CO_2_ continuous surging rate. Different work is done to solve this problem from time to time for gas sensitivity, and gas separation composites are made up of polymers and ILs. These ILs based on imidazolium are mainly used in the gas separation process because they are highly soluble in CO_2_, highly diffusible, and much permeable.^[^ ^318^ ^]^ Their infusibility and selectivity are greater than ammonium as well as ILs that were based on phosphonium. The H present that is acidic in the cationic part affects the carbon dioxide solubility of H‐bonding.^[^ ^319^ ^]^ Due to fluoride anions like PF_6_, BF_4_, and TFSI give much solubility in CO_2_ based compounds. These anions act as Lewis's base, and CO_2_ acts as Lewis's acid. Due to this, the CO_2_ and anions solubility increase.^[^ ^320^ ^]^ Poly(amide‐*b*‐ethylene oxide) (Pebax) acts as a good separating material for carbon dioxide from polluted air. Due to its high separation performance, Pebax 1657 is the commercially best Pebax. Polar components are present in both hard and soft segments of Pebax 1657; due to this, it has high solubility selectivity for CO_2_/H_2_ compared to other Pebax.^[^ ^321^ ^]^ The anion plays an essential part in the permeability of CO_2_. The introduction of IL also increases the affinity of NPs with the polymer matrix, primary to superior CO_2_/N_2_ selectivity. The result of [EMIM] [BF_4_] inclusion into Pebax 1657 is a hollow‐fiber gel membrane and based on Pebax, which is shown after the adding up of IL, after that a CO_2_ permeability was observed up to 300%. **Figure** **31**a,b illustrates the cross‐section SEM pictures of the compound hollow fibers. It is demonstrated the development of pressure upon permeability via TFC Pebax/IL gel membranes at 35 °C through initial pressure varying from 1 to 8 bar as displayed in Figure 31c–g.^[^ ^322^ ^]^

Nf–IL–SiO_2_ superhydrophobic membranes that are made from using nanocomposite, i.e., tetramethylammonium hydroxide IL, are utilized for separation of water vapor (WP). The composite membrane was used as another method for the separation of WP separation from flue gas.^[^ ^323^ ^]^

#### 4.5.3 Food Packaging

The development of materials from renewable sources is becoming a paramount and paradigmatic activity for the R&D and industrial communities. Significant work is undergoing to design sustainable and functional substances from biopolymers that may substitute traditional non‐biodegradable compounds.^[^ ^324^ ^]^ Despite all these steps, implementations like in biomedical tools, edible layers, and packings, bioactive sheets and functional packaging demand additionally breakthrough investigation.^[^ ^325^ ^]^ By the breakdown of collagen with water, a protein (gelatin) is produced. Gelatin also may produce flicks and covers through fine optical opaqueness and robotic potency.^[^ ^326^ ^]^ The actual content of aromatic amino acids has an intrinsic UV intercepting capability, creating universal materials.

Food packing is essential to enhance food protection and security. The evolution of novel, inventive packaging substances with antimicrobial effects may be accomplished through counting agents like antimicrobial ILs to hinder microbial development by facilitating live calculations of microbes.^[^ ^327^ ^]^ Green antimicrobial hybrid layers have been designed utilizing‐allyl‐3‐mthylimidazolium chloride and biopolymers, for example, cellulose, lignin, and starch, as qualified prospects for fresh food wrapping.^[^ ^328^ ^]^

**Figure 32:**
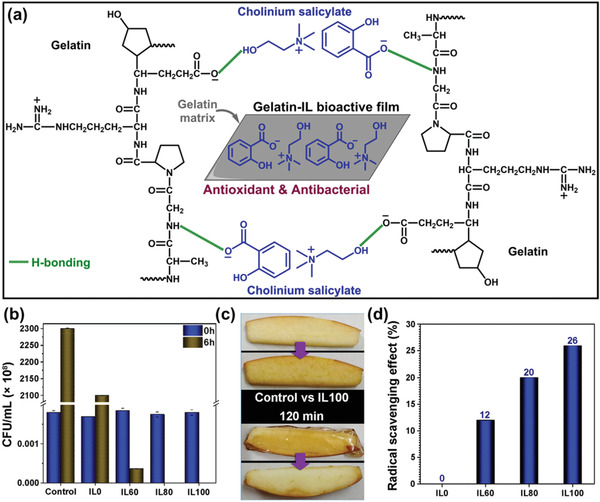
a) IL/gelatin‐based sheets and the H‐bonding relations among IL and gelatin in its configuration. b) Antibacterial development of prepared sheets anti to *Bacillus subtilis*. c) Examination of peeled apple slices’ protection. d) The free radical scavenging consequence is equipped with gelatin sheets’ diverse IL content. Reproduced with permission.^[^ ^329^ ^]^ Copyright 2021, Elsevier Ltd.

A bioactive IL, i.e., choline salicylate, was used to create gelatin‐based ion gel sheets (**Figure** **32**a) with improved antimicrobial and antioxidant characteristics. The IL served as a plasticizer within the film's design and did not participate in any chemical modifications during the preparation method. The IL/gelatin‐based sheets showed more bacterial development inhibition than the films in the absence of IL. It can be due to choline salicylate's good antibacterial impact anti to *Bacillus subtilis* (Figure 32b). The IL‐doped sheets also showed the capability to enhance the rack life of *Malus pumila* (red apple) via controlling oxidation in the air (Figure 32c); films showed excellent ultraviolet protection and antioxidant effects with higher mechanical stability. The consequences of antioxidant performance generated through free radical scavenging assay showed that the radical scavenging consequence did not appear in the sheet in the absence of IL. By contrast, the IL‐doped sheets with advanced IL concentration showed improved antioxidant capability (Figure 32d).^[^ ^329^ ^]^

In general, the application of ILs to design sheets for packing food is much restricted to bioactive or biopolymer‐based sheets. In the coming days, the investigation requires preferentially concentrated upon inventing dynamic, competent, and sturdy films and measures to improve protection by controlling film‐associated toxicity when revealed to the natural atmosphere. These textiles packaging sheets may be optimized and industrialized toward a superior position as dynamic and competent packaging for optical grade valuation of refreshed food outcomes.^[^ ^330^ ^]^ **Table** **5** shows the ILs‐based polymer composite in environmental applications.

**Table 5:** Ionic liquid‐based polymer composite in environmental applications

Sr. no.	ILs	Polymer materials	Applications	Refs.
1.	1‐Butyl‐3‐methyl imidazolium and bis(trifluoromethyl sulfonyl)imide[BMIM][TFSI]	Organic polymer (Pebax 1657)	Separation of CO_2_ from air	^[^ ^331^ ^]^
2.	1‐Ethyl‐3‐methylimidazoliumbis (trifluoromethylsulfonyl) imide[EMIM][TFSI]	Polysulfone (PSF)	CO_2_ capture	^[^ ^332^ ^]^
3.	1‐Butyl‐ 3‐methylimidazolium tetrafluoroborate[BMIM][BF_4_]	Polyethylene oxide (PEO)	CO_2_/N_2_ separation	^[^ ^333^ ^]^
4.	1‐Methyl‐3‐octylimidazolium hexanitratolanthanate[C_8_MIM]_3_[La(NO_3_)_6_] 1‐methyl‐ 3‐octylimidazolium pentanitratoeuropate [[C_8MIM_]_2_Eu(NO_3_)5)]	Polyvinyl pyrrolidone (PVP), polyvinyl alcohol (PVA), and polyacrylamide (PAM)	Adsorption particulate matter (PM) from air	^[^ ^334^ ^]^
5.	1‐Butyl‐3‐vinylimidazolium bromide[BVMIM][Br]	Vinyl polymer‐silica	Removal of phenolic pollutant from water	^[^ ^308^ ^]^
6.	1‐Methyimidazole and epichlorohydrin	Fe_3_O_4_–cellulose nanohybrid	Biosorption of Congo red dye from water	^[^ ^307^ ^]^
7.	1‐Aminoethyl‐3‐vinylimidazoliumchloride hydrochloride	Poly(divinylbenzene) [PDVB]	Removal of Cr(iv) impurities from water	^[^ ^311^ ^]^
8.	*N*‐methylimidazole based IL	DK110 resin	Adsorption of Acid Orange and Reactive Red from water	^[^ ^335^ ^]^
9.	1‐Methyimidazole	Cellulose	Removal of Congo red dye from water	^[^ ^336^ ^]^
10.	Dicationicionic liquid (DICAT)	Polyaniline (PANI), magnetic nanoparticles (MNPs)	Determination of polycyclic aromatic hydrocarbon in an environmental sample	^[^ ^337^ ^]^
11.	1‐Butyl‐3‐methylimidazolium bis(trifluoromethanesulfonyl)amide [C_4_mim][NTf_2_] or [C_4_mim][PF_6_]	Sulfonated polyimide (SIP)	CO_2_ separation	^[^ ^338^ ^]^
12.	Tetramethylammonium hydroxide (TMAOH)	Nf and mesoporous SiO_2_	Absorption of water vapor from the air	^[^ ^323^ ^]^
13.	1‐Carboxybutyl‐3‐methylimidazolium chloride	Chi	Anion adsorbent from wastewater	^[^ ^339^ ^]^
14.	[BMIM][Cl], [BMIM][BF_4_], [EOHMIM][Gly]	Polyurethanefoam	CO_2_ capture	^[^ ^340^ ^]^

### 4.6 ILs‐Based Polymer Nanocomposite for Aviation and Aerospace Industry Applications

The aviation and aerospace industry are one of the largest adopters of high‐performance composites, using 50% of the total production of advanced composites in the US. The motivation of using composites in aerospace is similar to the automotive and marine industries.^[^ ^341^ ^]^ The main concerns of in aerospace is the development of lightweight airframe at low cost and radiation‐proof for space applications. The weight reduction by using polymeric composites is a significant characteristic, because it also affects fuel effectiveness, aircraft speed, number of arranged parts, and range of aircraft. As stated by Koniuszewska and Kaczmar,^[^ ^342^ ^]^ by reducing the one‐pound weight of each aircraft, American airlines save 11 000 gallons of fuel every year on behalf of only 600 planes. Radiation protection of aircraft spacecraft can be achieved by using a polymer matrix reinforced with nanofillers in place of metal. The protecting efficiency of polymer composite materials (PCMs) is certified to their insulating property and the opportunity of manipulating them to have extensive‐Z fillers that are harmless and give advanced protection from X‐ray.^[^ ^343^ ^]^

The NPs based on carbon like graphene, CNT, and carbon black offer excellent aircraft protection from oxidation in the air.^[^ ^344^ ^]^ PCMs can also be utilized for various parts in aircraft like brakes, window frames, bulkheads, rotors, fuselage, brackets, wing boxes, fittings, airframe, blades, food tray arms, vertical fins, and tail assemblies due to their durability, much tolerance to atmospheric conditions, flexibility, corrosion resistance, with reduced noise level as well as resistance to fracture in nature.^[^ ^345^ ^]^ The aircraft's specific potency and rain erosion opposition can be enhanced by using hybrid kenaf/glass fiber FRPCs. On the other hand, temperature endurance can be increased by using carbon fiber reinforced silicon carbide in aircraft brakes as high as 1200 °C.

## 5 Current Challenges and Future Prospects

The published work demonstrates that various IL‐based adsorbents have been designed utilizing distinct synthesis approaches to complete the necessities of different sample practice technologies for extraction. Adsorbents that were based on IL, particularly IL‐based magnetic compound adsorbents, have been effectively used to initial‐concentrate all types of foodstuff pollutants into food samplings. Despite the significant improvement in preparing and applying IL‐based adsorbents to food research, a few issues must be managed. The primary issue is the shortage of IL‐based adsorbents, including positively discerning adsorption capability. Incorporating IL‐based MIPs have delivered a unique approach to designing IL‐based adsorbents, including elevated extractive selectivity.

Nevertheless, the numeral of ILs which may be utilized to design IL‐based MIPs is restricted to imidazole‐based ILs. Additionally, steps should be committed to developing the usage of new kinds of ILs to prepare MIPs. Similarly, compounds with other encouraging substances such as COFs may enhance the adsorption selectivity of IL‐based adsorbents. One more point is that IL‐based adsorbents toward food research are established explicitly upon IL‐based magnetic complexes.

Additionally, NP distributions and exterior functionalization, ILs and NPs may be incorporated within other composites to produce synergistic consequences. Colloidal gels are assembled while inconsistent dispersed NPs interconnect to create a 3D system, while colloidal glasses are acquired when interfacing NPs entangle the concentrated spread NPs. Additional NP and IL hybrid systems classes possess NP and IL‐based fluid crystalline catalysts and NP‐stabilized IL‐based emulsions. IL and NP combinations are usually incorporated within membranes or layers as covering composites toward electrodes, substrates solid‐phase composites, or gas partitions. Enhanced activity of the membranes has been completed the modification of NP and ILs subsequently. Membranes containing ILs and NPs have been broadly investigated to isolate gases. The examined more reasonable selectivity for gas detachments has been attributed to the interchanges among NP/IL and the response or separation solution. A unique synergistic impact of NP and the IL‐incorporated sheet was followed, improving the catalytic performance and stability due to establishing a more effective porous connection area among liquid and gas stages in the membrane network. The structure of the film permitted more ionic interchanges between the NP and various charged entities, leading to higher conductivity and better electrochemical and thermal steadiness. Electrochemical sensors with sheets simulated with ILs and NPs exhibited better sensibility and reaction.

Improved performance of the affinities between interactions, configuration, and effects of IL and NP combinations could enable the analytical method of such substances toward different applications. An optimized interpretation under specified working necessities may be executed for the preferred usage by carefully selecting NPs and ILs and their following community within a network which performs most suitable toward this distinct possibility. The applications of IL and NP have grown in current times, inspired by the improved effects of these substances. By encouraging implementations, additional investigation of the structure–property connections of NP and IL mixtures is balanced to enable the practical design of these novel substances and their conversion to research.

Further, to remarkably enhance the production of IL‐based polymeric materials, a design, including a high dissociation stable on low heat, must be developed. Few potential methods may be accepted, such as designing unique ILs and IL‐based monomers, hybrid ILs, and DESs possessing two or more ILs and employing these within the polymeric chain. We expect this perspective to understand ion gels and their implementations better, and lock the hole among the numerous corresponding areas. Furthermore, to the LIBs and SCs discussed within this article, it is envisaged that IL investigation advancements will be used in the forthcoming ESS and transformation appliances, for example, polyvalent ion batteries, metal–air batteries, and competent fuel cells.

Due to their distinctive characteristics and nature, ILs have been growing as candidates for advanced materials. The development of novel ILs depends upon the choice of their cations and anions and the choice of the polymer matrix, which leads to the formation of work specific ILs with extraordinary characteristics. For the use in various fields, ILs/polymers can be modified into various forms like gel, fibers, films, and accordingly, their properties also changed. This discussion is ongoing as we are moving toward developing green chemistry, but volarization, i.e., separation and oxidation of lignin, is an important challenge. To overcome this problem, lignin dissolved into the ILs and was extracted. The sulphonate and phosphate anion based ILs selectively dissolve the lignin and oxidize it to give more valuable renewable chemicals.^[^ ^346^ ^]^ Further ILs also used in the treatment of waste water^[^ ^293^ ^]^ in which many ILs are used, so in the future; we need to develop some methods for the regeneration of ILs from wastewater for further use and development of nontoxic ILs.

## 6 Conclusion

IL‐based adsorbents and electrolytes have considerably drawn concentration within the polymeric material synthesis area in current years. In this study, ILs based polymer composites and their exceptional physicochemical characteristics their capability to bond by analytes via multiple interchanges, such as *π*–*π*, hydrogen interaction, ion‐exchange, electrostatic, and dipolar relations, have been discussed in detail. Different IL and polymer‐based NP combinations have been discussed based on a proportion of numerous intermolecular relations. These combinations may incorporate unexplored effects of NPs and ILs, which help possible implementations. This article discussed synergistic consequences of IL and polymer‐based NP hybrids in the context of appearing applications of such mixtures within the domains of catalysis, biomedicine, environmental, actuators, electrochemistry, and purifications.

Different configurations of IL and polymer‐based NP mixtures have been used to sufficiently sustain these applications. Steady NP distributions within ILs may be executed while repulsive intermolecular relations are more robust than stunning intermolecular synergies among neighboring NPs. The IL may comprise a coating near the NPs to avoid the accumulation and/or corrosion that enhances the operational efficiency of apparatuses or grows their activity under extreme working circumstances. Rationally developed MNP and IL‐based compounds have been used as positively capable materials in various phase catalytic responses. The better proton‐transfer effects of ILs and the increased electronic performance of MNPs distributions within ILs have been employed in electrochemical usages like batteries and electrochemical‐based sensors. Incorporating ILs‐based NPs in electrolytes toward energy storage material has been demonstrated to enhance the cations diffusion coefficient within the IL solution, electrical activity, thermal and electrochemical strength, and decrease corrosion. The accumulation of NPs, including catalytic effects, may reduce the overpotentials of multiple electrochemical responses, contributing to susceptible electrochemical sensor strategies. Still, there is room to develop a rational design for the fabrication of ILs‐based polymer nanocomposites that could provide advanced methods without compromising the extensive properties of ILs and could harness them in the end product.

## Conflict of Interest

The authors declare no conflict of interest.
